# Supplementary material for: Identification of Putative Vaccine and Drug Targets against the Methicillin-Resistant Staphylococcus aureus by Reverse Vaccinology and Subtractive Genomics Approaches
Source: Molecules. 2022 Mar 24;27(7):2083. doi: 10.3390/molecules27072083 (PMC9000511; doi:10.3390/molecules27072083)
Supplement: Supplementary file 1 [file molecules-27-02083-s001.zip › molecules-1586608-supplementary materials.pdf]

molecules

MDPI

Supplementary files

Table S1. Features of sixteen whole-genome sequences of methicillin-resistant *S. aureus* strains used for the identification of vaccine candidates and drug target proteins

Strain ID	MLST Type	Clonal complex (CC)	Size (bp)	GC%	CDS	Accession No.
SA G5	ST45	CC45	2760385	32.77	2689	CP032160
SA G6	ST228	CC5	2856214	32.79	2734	RAHA00000000
SA G8	ST225	CC8	2857863	32.81	2743	QZFC00000000
SA H27	ST22	CC22	2783185	32.73	2630	CP032161
SA H29	ST8	CC8	2834624	32.65	2843	CP032468
SA H32	ST22	CC22	2786627	32.72	2657	RAHP00000000
NCTC 8325	ST8	CC8	2821361	32.9	2872	CP000253.1
CA-347	ST45	CC45	2875156	32.9	2766	CP006044.1
ST228	ST228	CC5	2783086	32.82	2654	HE579071.1
JH9	ST105	CC5	2937129	32.9	2879	CP000703.1
Newman	ST254	CC8	2878897	32.89	2854	AP009351.1
HO 5096 0412	ST22	CC22	2832299	32.8	2527	HE681097.1
Mu50 DNA	ST5	CC5	2878529	32.87	2867	BA000017.4
MRSA252	ST36	CC30	2902619	32.80	2872	BX571856.1
H-EMRSA-15	ST22	CC22	2846320	32.80	2740	CP007659
DSM 20231	ST8	CC8	2755072	32.88	2734	CP011526.1

Table S2. Subtractive genome analysis of methicillin-resistant *S. aureus* strains

Features	Number of proteins
Core-genome	1719
Non-redundant proteins	1678
Essential core-proteins	278
Essential non-paralogous proteins	98
Cytoplsamic (CYT) proteins	78
Secreted (SEC) proteins	2
Potentially surface exposed (PSE) proteins	6
Membrane (MEM) proteins	12

Table S3. Linear B-cell epitopes of 4 putative vaccine candidates of *S. aureus* determined by SVNTrip (20mers)

Protein Name	Linear B-cell epitopes	Start-End	Score
Foldase protein (PrsA)	DTMKKIGKDQIANASFTEML	45 - 64	1.000
	GEVSDVVKSSFGYHIIKADK	226 - 245	0.438
	KMINKLIVPVTASALLLGAC	2 - 21	0.366
	DFKDRDIKSVVEDKILNPEK	286 - 305	0.359
	YKENLRTAAYHKELLSDKIK	112 - 131	0.222
ESAT-6 machinery protein (EssA)	LTFLTASSNNGGLNIDVQQE	9 - 28	1.000
Penicillin-binding protein 1 (PBP1)	YSSNTLMMHLQDLVGADKMK	367 - 386	1.000
	TYQDKLKIEKMNLPGISLLP	146 - 165	0.603
	STFKSYGLAAAIQEGAFDPD	314 - 333	0.502
	QSIKAGNKVLPHSKVLLLTD	636 - 655	0.442
	KNKIGAVLLVGLFGLLFFIL	10 - 29	0.410
	AFKPIMENTLKYLNVGKSKD	573 - 592	0.403
	KGSGFVSHQSISKGQKLTEK	685 - 704	0.353
	FNPETGKDFGKKWANDLYQN	289 - 308	0.343
	KDLFAVVMDAKTGEILAYSQ	266 - 285	0.336
	VERYKLVAVIDKKASANSKK	80 - 99	0.303
DD-transpeptidase (PBP2)	TSHKAMSDYTAYMLAEMLKG	543 - 562	1.000
	KAQEAYLSYRLEQEYSKDDI	168 - 187	0.564
	ENVMSKISSRDGEDFKRPSS	649 - 668	0.425
	YKRLFGAIGKNLTGGFGSEG	126 - 145	0.377
	WEDAKKIDLKANLVNRTAEE	268 - 287	0.372
	KNKDQQVGATILDSKTGGLV	354 - 373	0.332
	KAAEDRKNTVLYLMHYHKRI	244 - 263	0.306
	YDKNGELVKTLDNGQRHEHV	78 - 97	0.290
	FKDENLGNVLQSGIKIYTNM	316 - 335	0.286
	GFSKVKQYGENSFVGHSQQE	623 - 642	0.263

Table S4. Drug target prioritization parameters and functional analysis of eight protein targets.

**Protein Name**	**Protein ID***	**Gene**	**Length (aa)**	**Mo. Wt. (KDa)**	**Structural quality MHOLline**	**Function**
Biotin protein ligase	WP_000049913.1	*birA*	323	37.07	Very High	**MF**: Biotin-[acetyl-CoA-carboxylase] ligase activity. **BF**: Protein biotinylation
HPr kinase/phosphorylase	WP_000958224.1	*hprK*	310	34.48	Very High	**MF:** ATP binding Source, magnesium ion binding, phosphorelay sensor kinase activity, protein serine/threonine/tyrosine kinase activity, protein serine/threonine kinase activity. **BF:** Carbohydrate metabolic process, regulation of carbohydrate metabolic process
Thymidylate kinase	WP_001272126.1	*tmk*	210	24.06	Very High	**MF:** Thymidylate kinase activity. **BF:** Nucleotide biosynthesis
Phosphate acetyltransferase	WP_000774281.1	*pta*	328	34.98	Very High	**MF:** Phosphate acetyltransferase activity, **BF:** Acetyl-CoA biosynthetic process
UDP-N-acetylmuramoyl-L-alanyl-D-glutamate--L-lysine ligase	WP_000340119.1	*murE*	494	54.21	Very High	**MF:** UDP-N-acetylmuramoyl-L-alanyl-D-glutamate-L-lysine ligase activity. **BF:** Cell cycle, Cell division, Cell shape, Cell wall biogenesis/degradation, Peptidoglycan synthesis
UTP--glucose-1-phosphate uridylyltransferase	WP_000721337.1	*gtaB*	288	32.49	Very High	**MF:** UTP:glucose-1-phosphate uridylyltransferase activity. **BF:** Enterobacterial common antigen biosynthetic process, UDP-glucose metabolic process
Fatty acid/phospholipid synthesis	WP_000239744.1	*plsX*	328	35.43	High	**MF:** Phosphate:acyl-[acyl carrier protein] acyltransferase activity. **BF:** Fatty acid biosynthetic process, phospholipid biosynthetic process
Pantothenate synthetase	WP_000163742.1	*panC*	283	31.44	Good	**MF:** ATP binding, pantoate-beta-alanine ligase activity. **BF:** Pantothenate biosynthesis

Protein ID represent the NCBI protein accession number, **MF** and **BF** represent Molecular function and biological function, respectively of the identified target proteins

Table S5. Identified druggable pocket with its volume, surface area and drug score of each target protein obtained from DoGSiteScorer.

Target protein	Volume (Å³)	Surface area (Å²)	Drug score	Binding site residues
Biotin protein ligase	1425.15	1648.25	0.80	SER93, THR94, GLN95,LEU96, LYS99, LN116, GLY119, ARG120, GLY121, ARG122, ARG125, HIS126, TRP127, SER128, SER129, SER130, GLN133, GLY134, LEU135, TRP136, MET137, SER138, SER148, MET149, ILE150, SER151, LYS152, ASN154, LEU155, ALA158, VAL175, LYS176, TRP177, PRO178, ASN179, ASP180, TYR182, LYS187, VAL188, GLY190, PHE191, LEU192, THR193, GLU194, GLY208, ILE209, GLY210, ASN212, GLN215, ASP219, PHE220, ILE224, ARG227, ALA228, TYR271, ASP301, GLY302, TYR303, LEU316, ILE317, SER318, ALA319, ASP320, ILE321, ASP322
HPr kinase/phosphorylase	562.79	751.65	0.77	VAL147, LEU148, ILE149, THR150, GLY151, ASP152, ILE155, LYS157, GLU159, THR160, GLU163, LEU226, ASN227, ILE228, ASN229, LYS258, LYS259, THR260, ILE261, VAL263, ARG267, ASN268, VAL269, ALA270, ILE273, ALA276
Thymidylate kinase	470.78	479.38	0.73	ARG41, GLU42, PRO43, GLY49, ILE52, ARG53, VAL56, GLU67, ALA68, MET69, LEU70, PHE71, ALA72, SER74, ARG75, CYS95, ASP96, ARG97, TYR98, SER101, SER102, TYR105, GLN106, VAL117, LEU120, ASN121
Phosphate acetyltransferase	415.87	484.65	0.77	LYS143, THR149, ILE152, PHE153, PHE154, ILE163, GLU184, LYS187, SER188, SER191, PHE192, VAL292, VAL295, LEU296, GLN297, GLY298, LEU299, ASN300, SER301, PRO302, VAL303, ASN304, ILE321, GLN325, ALA326, LEU327, GLN328
UDP-N-acetylmuramoyl-L-alanyl-D-glutamate-L-lysine ligase	703.09	912.87	0.85	THR115, ALA118, THR119, HIS122, LEU123, ARG126, LYS127, TYR134, ASN138, GLY139, PHE140, GLN141, ASN143, GLU144, LYS146, THR147, GLU330, PRO331, VAL332, GLU333
UTP-glucose-1-phosphate uridylyltransferase	430.21	506.94	0.66	GLY109, LEU110, GLY111, LEU130, ASP132, ASP133,TYR170, GLY171,PHE188, VAL189, GLU190, LYS191,

				ILE203, MET204, GLY205, TYR207, ASN226, ILE228, GLN229, LEU230, THR231, ILE234, ARG252, ASP254, GLY256
Fatty acid/phospholipid synthesis	2202.2	2922.41	0.82	MET9, GLY11, ASP12, ASN13, PRO15, ILE59, GLU60, MET61, GLU62, MET77, SER94, ALA95,GLY96, ASN97, THR98, ALA100, LEU101, ILE112, VAL115, ALA119, LEU120, VAL121, VAL122, THR123, LEU124, PRO125, THR126, ILE127, ASP128, GLY129, LYS130, GLY131, PHE132, VAL133, PHE134, LEU135, VAL137, GLN150, GLN153, LEU154, ILE157, TYR158, ILE162, ARG163, ALA207, LYS208, LEU210, MET211, GLY225, ASN226, VAL228, LEU229, ASN231,LEU232, GLU233, THR235, ALA236, ALA258, ILE259, LEU260, LYS261, LYS262, ASP263, LEU264, ALA265, GLU266, PHE267, ALA268, LYS269, LYS270, MET271, ASP272, TYR273, TYR276, GLY277, GLY278, SER279, VAL280, LEU281, LEU282, HIS292, SER295, ASN296, LYS298, ALA299, TYR301, ALA303, ILE304, ALA307, ALA310, GLY311
Pantothenate synthetase	1400.83	1071.11	0.81	ILE28, PRO29, THR30, MET31, GLY32, HIS35, GLY37, HIS38, THR40, MET41, SER55, VAL56, PHE57, VAL58, ASN59, GLN62, PHE63, TYR72, PHE92, PRO94, MET99, LEU117, GLU118, ARG122, HIS125, PHE126, VAL129, VAL132, VAL133, LEU136, TYR146, PHE147, GLY148, LYS149, LYS150, ASP151, ALA152, GLN153, GLN154, ILE174, ASP175, ILE176, VAL177, ARG178, GLY182, LEU183, ALA184, LYS185, SER186, SER187, ARG188, ASN189, ALA200, VAL201, HIS202, LEU203, SER204, GLU242, PHE262, SER264, LEU265, ALA266, PHE269, ARG273, LEU274, ILE275, ASP276, ASN277

*Amino acid with red color represent the amino acid residues involved in H-bond formation with respective ligand.

Sequence file 1: Human_Non-Homologous_Essential_protein sequences

>10 chromosomal replication initiator protein DnaA
MSEKEIWEKVLEIAQEKLSAVSYSTFLKDTELYTIKDGEAIVLSSIPFNANWLNQQYAEIIQAILFDVVGYEVKPHFITTEELANYSNNETATPKETTKPST
ETTEDNHVLGREQFNAHNTFDTFVIGPGNRFPHAASLAVAEAPAKAYNPLFIYGGVGLGKTHLMHAIGHHVLDNNPDAKVIYTSSEKFTNEFIKSIR
DNEGEAFRERYRNIDVLLIDDIQFIQNKVQTQEEFFYTFNELHQNNKQIVISSDRPPKEIAQLEDRLRSRFEWGLIVDITPPDYETRMAILQKKIEEEKLD
IPPEALNYIANQIQSNIRELEGALTRLLAYSQLLGKPITTELTAEALKDIIQAPKSKKITIQDIQKIVGQYYNVRIEDFSAKKRTKSIAYPRQIAMYLSRELTD
FSLPKIGEEFGGRDHTTVIHAHEKISKDLKEDPIFKQEVENLEKEIRNV

>20 DNA polymerase III, beta chain
MMEFTIKRDYFITQLNDTLKAISPRTTLPILTGIKIDAKEHEVILTGSDSEISIEITIPKTVDGEDIVNISETGSVVLPGRFFVDIIKKLPGKDVKLSTNEQFQT
LITSGHSEFNLSGLDPDQYPLLPQVSRDDAIQLSVKVLKNVIAQTNFAVSTSETRPVLTGVNWLIQENELICTATDSHRLAVRKLQLEDVSENKNVIIPG
KALAELNKIMSDNEEDIDIFFASNQVLFKVGNVNFISRLLEGHYPDTTRLFPENYEIKLSIDNGEFYHAIDRASLLAREGGNNVIKLSTGDDVVELSSTSP
EIGTVKEEVDANDVEGGNLKISFNSKYMMDALKAIDNDEVEVEFFGTMKPFILKPKGDDSVTQLILPIRTY

>DNA gyrase A subunit
LKEELLMAELPQSRINERNITSEMRESFLDYAMSVIVARALPDVRDGLKPVHRRILYGLNEQGMTPDKSYKKSARIVGDVMGKYHPHGDSSIYEAMV
RMAQDFSYRYPLVDGQGNFGSMDGDGAAAMRYTEARMTKITLELLRDINKDTIDFIDNYDGNEREPSVLPARFPNLLANGASGIAVGMATNIPPH
NLTELINGVLSLSKNPDISIAELMEDIEGPDFPTAGLILGKSGIRRAYETGRGSIQMRSRAVIEERGGGRQRIVVTEIPFQVNKARMIEKIAELVRDKKID
GITDLRDETSLRTGVRVVIDVRKDANASVILNNLYKQTPLQTSFGVNMIALVNGRPKLINLKEALVHYLEHQKTVVRRRTQYNLRKAKDRAHILEGLRI
ALDHIDEIISTIRESDTDKVAMESLQQRFKLSEKQAQAILDMRLRRLTGLERDKIEAEYNELLNYISELEAILADEEVLLQLVRDELTEIRDRFGDDRRTEI
QLGGFENLEDEDLIPEEQIVITLSHNNYIKRLPVSTYRAQNRGGRGVQGMNTLEEDFVSQLVTLSTHDHVLFFTNKGRVYKLKGYEVPELSRQSKGIP
VVNAIELENDEVISTMIAVKDLESEDNFLVFATKRGVVKRSALSNFSRINRNGKIAISFREDDELIAVRLTSGQEDILIGTSHASLIRFPESTLRPLGRTAT
GVKGITLREGDEVVGLDVAHANSVDEVLVVTENGYGKRTPVNDYRLSNRGGKGIKTATITERNGNVVCITTVTGEEDLMIVTNAGVIIRLDVADISQ
NGRAAQGVRLIRLGDDQFVSTVAKVKEDAEDETNEDEQSTSTVSEDGTEQQREAVVNDETPGNAIHTEVIDSEENDEDGRIEVRQDFMDRVEEDI
QQSSDEE

>SAEMRSA100160 DnaB-like helicase
MDRMYEQNQMPHNNEAEQSVLGSIIIDPELINTTQEVLLPESFYRGAHQHIFRAMMHLNEDNKEIDVVTLMDQLSSEGTLNEAGGPQYLAELSTN
VPTTRNVQYYTDIVSKHALKRRLIQTADSIANDGYNDELELDAILSDAERRILELSSSRESDGFKDIRDVLGQVYETAEELDQNSGQTPGIPTGYRDLD
QMTAGFNRNDLIILAARPSVGKTAFALNIAQKVATHEDMYTVGIFSLEMGADQLATRMICSSGNVDSNRLRTGTMTEEDWSRFTIAVGKLSRTKIFI
DDTPGIRINDLRSKCRRLKQEHGLDMIVIDYLQLIQGSGSRASDNRQQEVSEISRTLKALARELECPVIALSQLSRGVEQRQDKRPMMSDIRESGSIEQ
DADIVAFLYRDDYYNRGGDEDDDDDGGFEPQTNDENGEIEIIIAKQRNGPTGTVKLHFMKQYNKFTDIDYAHADMM

>SAEMRSA100180 response regulator protein
MQMARKVVVVDDEKPIADILEFNLKKEGYDVYCAYDGNDAVDLIYEEEPDIVLLDIMLPGRDGMEVCREVRKKYEMPIIMLTAKDSEIDKVLGLELG
ADDYVTKPFSTRELIARVKANLRRHYSQPAQDTGNVTNEITIKDIVIYPDAYSIKKRGEDIELTHREFELFHYLSKHMGQVMTREHLLQTVWGYDYFG
DVRTVDVTIRRLREKIEDDPSHPEYIVTRRGVGYFLQQHE

>SAEMRSA103150 DNA-binding protein
VSELSKSEDQRITKTKYEQIKQIDISDIKPNPYQPRKTFDENHLNDLADSIKQYGILQPIVLRKTVQGYYIVVGERRFRASKIAGLKYVSAIIKDLTDEDM
MELAVIENLQREDLNAIEEAESYQRLMTDLKITQQEVAKRLSKSRPYIANMLRLLHLPKKIADMVKDGRLTSAHGRTLLAIKDEQQMLRLAKRVVKEK
WSVRYLENHVNELKNVSSKSETDKVDITKPKFIKQQERQLREQYGAKVDISIKKSVGKISFEFESQDDFVRIIEQLNRKYGK

>SAEMRSA103210 putative single strand DNA-binding protein
MLNRVVLVGRLTKDPEYRTTPSGVSVATFTLAVNRTFTNAQGEREADFINCVVFRRQADNVNNYLSKGSLAGVDGRLQSRNYENQEGRRVFVTEV
VCDSVQFLEPKNAQQNGGQRQQNEFQDYGQGFGGQQSGQNNSYNNSSNTKQSDNPFANANGPIDISDDDLPF

>NWMN 0446 thymidylate kinase
LRMNKMSAFITFEGPEGSGKTTVINEVYHRLVKDYDVIMTREPGGVPTGEEIRKIVLEGNDMDIRTEAMLFAASRREHLVLKVIPALKEGKVVLCDRY
IDSSLAYQGYARGIGVEEVRALNEFAINGLYPDLTIYLNVSAEVGRERIIKNSRDQNRLDQEDLKFHEKVIEGYQEIIHNESQRFKSVNADQPLENVVE
DTYQTIIKYLEKI

>SAEMRSA104210 conserved hypothetical protein

MIYETAPAKINFTLDTLFKRNDGYHEIEMIMTTVDLNDRLTFHKRKDRKIVVEIEHNYVPSNHKNLAYRAAQLFIEQYQLKQGVTISIDKEIPVSAGLA
GGSADAAATLRGLNRLFDIGASLEELALLGSKIGTDIPFCIYNKTALCTGRGEKIEFLNKPPSAWVILAKPNLGISSPDIFKLINLDKRYDVHTKMCYEALE
NRDYQQLCQSLSNRLEPISVSKHPQIDKLKNNMLKSGADGALMSGSGPTVYGLARKESQAKNIYNAVNGCCNEVYLVRLLG

>SAI7S6 1003710 50S ribosomal protein L25
MRMASLKSIIRQGKQTRSDLKQLRKSGKVPAVVYGYGTKNVSVKVDEVEFIKVIREVGRNGVIELGVGSKTIKVMVADYQFDPLKNQITHIDFLAIN
MSEERTVEVPVQLVGEAVGAKEGGVVEQPLFNLEVTATPDNIPEAIEVDITELNINDSLTVADVKVTGDFKIENDSAESVVTVVAPTEEPTEEEIEAM
EGEQQTEEPEVVGESKEDEEKTEE

>SAEMRSA104350 conserved hypothetical protein
MQLNSNGWHVDDHIVVAVSTGIDSMCLLYQLLNDYKDSYRKLTCLHVNHGVRSASIEEARFLEVYCERHHIDLHIKKLDLSHSLDRNNSIQNEARIKR
YEWFDEMMNVLEADVLLTAHHLDDQLETIMYRIFNGKSTRNKLGFDELSKRKGYQIYRPLLAVSKKEIKQFQERYHIPYFEDESNKDNKYVRNDIRN
RIIPAIDENNQLKVSHLLKLKQWHDEQYDILQYSAKQFIQEFVKFDEQSKYLEVSRQAFNNLPNSLKMVVLDCLLSKYYELFNISAKTYEEWFKQFSSK
KAQFSINLTDKWIIQIAYGKLIIMAKNNGDTYFRVQTIEKPGNYIFNKYRLEIHSNLPKCLFPLTVRTRQSGDTFKLNGRDGYKKVNRLFIDCKVQQWV
RDQMPIVLDKQQRIIAVGDLYQQQTIKQWIIISKNGDE

>SAI7S6 1003810 Dihydropteroate synthase
MTKTKIMGILNVTPDSFSDGGKFNNVETAINRVKAMIDEGADIIDVGGVSTRPGHEMVTLEEEELNRVLPVVEAIVGFDVKISVDTFRSEVAEACLKL
GVDMINDQWAGLYDHRMFQIVAKYDAEIILMHNGNGNRDEPVVEEMLTSLLAQAHQAKIAGIPSNKIWLDPGIGFAKTRNEEAEVMARLDELVA
TEYPVLLATSRKRFTKEMMGYDTTPVERDEVTAATTAYGIMKGVRAVRVHNVELNAKLAKGIDFLKENENARHNLS

>SAEMRSA104630 transcription antitermination protein
MSEEVGAKRWYAVHTYSGYENKVKKNLEKRVESMNMTEQIFRVVIPEEEENQVKDGKAKTTVKKTFPGYVLVELIMTDESWYVVRNTPGVTGFV
GSAGAGSKPNPLLPEEVRFILKQMGLKEKTIDVELEVGEQVRIKSGPFANQVGEVQEIETDKFKLTVLVDMFGRETPVEVEFDQIEKL

>SAEMRSA104650 50S ribosomal protein L1
MAKKGKKYQEAASKVDRTQHYSVEEAIKLAKETSIANFDASVEVAFRLGIDTRKNDQQIRGAVVLPNGTGKSQSVLVFAKGDKIAEAEAAGADYVG
EAEYVQKIQQGWFDFDVVVATPDMMGEVGKLGRVLGPKGLMPNPKTGTVTMDVKKAVEEIKAGKVEYRAEKAGIVHASIGKVSFTDEQLIENFN
TLQDVLAKAKPSSAKGTYFKSVAVTTTMGPGVKIDTASFK

>SAEMRSA104660 50S ribosomal protein L10
MSAIIEAKKQLVDEIAEVLSNSVSTVIVDYRGLTVAEVTDLRSQLREAGVEYKVVKNTMVRRAAEKAGIEGLDEFLTGPTAIATSSEDAVAAAKVISGF
AKDHEALEIKSGVMEGNVITAEEVKTVGSLPSHDGLVSMLLSVLQAPVRNFAYAVKAIGEQKEESAE

>SAEMRSA105160 putative phosphate acetyltransferase
MADLLNVLKDKLSGKNVKIVLPEGEDERVLTAATQLQATDYVTPIVLGDETKVQSLAQKLNLDISNIELINPATSEFKAELVQSFVERRKGKATEEQAQ
ELLNNVNYFGTMLVYAGKADGLVSGAAHSTGDTVRPALQIIKTKPGVSRTSGIFFMIKGDEQYIFGDCAINPELDSQGLAEIAVESAKSALSFGMDPK
VAMLSFSTKGSAKSDDVTKVQEAVKLAQQKAEEEKLEAIIDGEFQFDAAIVPGVAEKKAPGAKLQGDANVFVFPSLEAGNIGYKIAQRLGGYDAVG
PVLQGLNSPVNDLSRGCSIEDVYNLSIITAAQALQ

>SAEMRSA106090 putative undecaprenol kinase
MFIIELIKGIILGVVEGLTEFAPVSSTGHMILVDDMWLKSSEFLGSQSAFTFKIVIQLGSVFAAAWVFRERFLEILHIGKHKHVEGENDQQRRSKPRRLN
LLHVLVGMVPAGILGLLFDDFIEEHLFSVPTVMIGLFVGAIYMIIADKYSAKVKNPQTVDQINYFQAFVIGISQAVAMWPGFSRSGSTISTGVLMKLN
HKAASDFTFIMAVPIMLAASGLSLLKHYQDIQIADIPFYILGFLAAFTVGLIAIKTFLHLINKIKLIPFAIYRIVLVIFIAILYFGFGIGKGI

>SAEMRSA106580 ribonucleoside-diphosphate reductase beta chain
MIAVNWNTQEDMTNMFWRQNISQMWVETEFKVSKDIASWKTLSEAEQDTFKKALAGLTGLDTHQADDGMPLVMLHTTDLRKKAVYSFMAM
MEQIHAKSYSHIFTTLLPSSETNYLLDEWVLEEPHLKYKSDKIVANYHKLWGKEASIYDQYMARVTSVFLETFLFFSGFYYPLYLAGQGKMTTSGEIIRK
ILLDESIHGVFTGLDAQHLRNELSESEKQKADQEMYKLLNDLYLNEESYTKMLYDDLGITEDVLNYVKYNGNKALSNLGFEPYFEEREFNPIIENALDT
TTKNHDFFSVKGDGYVLALNVEALQDDDFVFDNK

>SAEMRSA106640 putative UDP-N-acetylenolpyruvoylglucosamine reductase
VINKDIYQALQQLIPNEKIKVDEPLKRYTYTKTGGNADFYITPTKNEEVQAVVKYAYQNEIPVTYLGNGSNIIIREGGIRGIVISLLSLDHIEVSDDAIIAGS
GAAIIDVSRVARDYALTGLEFACGIPGSIGGAVYMNAGAYGGEVKDCIDYALCVNEQGSLIKLTTKELELDYRNSIIQKEHLVVLEAAFTLAPGKMTEI

QAKMDDLTERRESKQPLEYPSCGSVFQRPPGHFAGKLIQDSNLQGHRIGGVEVSTKHAGFMVNVDNGTATDYENLIHYVQKTVKEKFGIELNREV
RIIGEHPKES

>SAEMRSA106790 preprotein translocase SecA subunit
MGFLSKILDGNNKEIKQLGKLADKVIALEEKTAILTDEEIRNKTKQFQTELADIDNVKKQNDYLDKILPEAYALVREGSKRVFNMTPYKVQIMGGIAIH
KGDIAEMRTGEGKTLTATMPTYLNALAGRGVHVITVNEYLSSVQSEEMAELYNFLGLTVGLNLNSKTTEEKREAYAQDITYSTNNELGFDYLRDNM
VNYSEDRVMRPLHFAIIDEVDSILIDEARTPLIISGEAEKSTSLYTQANVFAKMLKQDEDYKYDEKTKAVHLTEQGADKAERMFKVENLYDVQNVDVI
SHINTALRAHVTLQRDVDYMVVDGEVLIVDQFTGRTMPGRRFSEGLHQAIEAKEGVQIQNESKTMASITFQNYFRMYNKLAGMTGTAKTEEEEFR
NIYNMTVTQIPTNKPVQRNDKSDLIYISQKGKFDAVVEDVVEKHKAGQPVLLGTVAVETSEYISNLLKKRGIRHDVLNAKNHEREAEIVAGAGQKGA
VTIATNMAGRGTDIKLGEGVEELGGLAVIGTERHESRRIDDQLRGRSGRQGDKGDSRFYLSLQDELMIRFGSERLQKMMSRLGLDDSTPIESKMVS
RAVESAQKRVEGNNFDARKRILEYDEVLRKQREIIYNERNSIIDEEDSSQVVDAMLRSTLQRSINYYINTADDEPEYQPFIDYINDIFLQEGDITEDDIKG
KDAEDIFEVVWAKIEAAYQSQKDILEEQMNEFERMILLRSIDSHWTDHIDTMDQLRQGIHLRSYAQQNPLRDYQNEGHELFDIMMQNIEEDTCKFI
LKSVVQVEDNIEREKTTEFGEAKHVSAEDGKEKVKPKPIVKGDQVGRNDDCPCGSGKKFKNCHGK

>SAEMRSA106860 HPr kinase/phosphatase
MLTTEKLVETLKLDLIAGEEGLSKPIKNADISRPGLEMAGYFSHYASDRIQLLGTTELSFYNLLPDKDRAGRMRKLCRPETPAIIVTRGLQPPEELVEAA
KELNTPLIVAKDATTSLMSRLTTFLEHALAKTTSLHGVLVDVYGVGVLITGDSGIGKSETALELVKRGHRLVADDNVEIRQINKDELIGKPPKLIEHLLEIR
GLGIINVMTLFGAGSILTEKRIRLNINLENWNKQKLYDRVGLNEETLSILDTEITKKTIPVRPGRNVAVIIEVAAMNYRLNIMGINTAEEFSERLNEEIIKN
SHKSEE

>SAR081prolipoprotein diacylglyceryl transferase
MGGIVFNYIDPVAFNLGPLSVRWYGIIIAVGILLGYFVAQRALVKAGLHKDTLVDIIFYSALFGFIAARIYFVIFQWPYYAENPGEIIKIWHGGIAIHGGLI
GGFIAGVIVCKVKNLNPFQIGDIVAPSIILAQGIGRWGNFMNHEAHGGPVSRAFLEQLHLPNFIIENMYINGQYYHPTFLYESIWDVAGFIILVNIRKH
LKLGETFFLYLTWYSIGRFFIEGLRTDSLMLTSNIRVAQLVSILLILISISLIVYRRIKYNPPLYSKVGALPWPTRKVK

>SAEMRSA107480 conserved hypothetical protein
MAKKAPDVGDYKYGFHDDDVSIFRSERGLTENIVREISNMKNEPEWMLDFRLKSLKLFYKMPMPQWGGDLSELNFDDITYYVKPSEQAERSWDE
VPEEIKRTFDKLGIPEAEQKYLAGVSAQYESEVVYHNMEKELEEKGIIFKDTDSALQENEELFKKYFASVVPAADNKFAALNSAVWSGGSFIYVPKNIK
LDTPLQAYFRINSENMGQFERTLIIADEGASVHYVEGCTAPVYTTSSLHSAVVEIIVHKDAHVRYTTIQNWANNVYNLVTKRTFVYENGNMEWVDG
NLGSKLTMKYPNCVLLGEGAKGSTLSIAFAGKGQVQDAGAKMIHKAPNTSSTIVSKSISKNGGKVIYRGIVHFGRKAKGARSNIECDTLILDNESTSDT
IPYNEVFNDQISLEHEAKVSKVSEEQLFYLMSRGISEEEATEMIVMGFIEPFTKELPMEYAVEMNRLIKFEMEGSIG

>SAEMRSA108480 UDP-N-acetylmuramoylalanyl-D-glutamate--2,6-dia minopimelate ligase
LDASTLFKKVKVKRVLGSLEQQIDDITTDSRTAREGSIFVASVGYTVDSHKFCQNVADQGCKLVVVNKEQ
SLPANVTQVVVPDTLRVASILAHTLYDYPSHQLVTFGVTGTNGKTSIATMIHLIQRKLQKNSAYLGTNGF
QINETKTKGANTTPETVSLTKKIKEAVDAGAESMTLEVSSHGLVLGRLRGVEFDVAIFSNLTQDHLDFHG
TMEAYGHAKSLLFSQLGEDLSKEKYVVLNNDDSFSEYLRTVTPYEVFSYGIDEEAQFMAKNIQESLQGVS
FDFVTPFGTYPVKSPYVGKFNISNIMAAMIAVWSKGTSLETIIKAVENLEPVEGRLEVLDPSLPIDLIID
YAHTADGMNKLIDAVQPFVKQKLIFLVGMAGERDLTKTPEMGRVACRADYVIFTPDNPANDDPKMLTAEL
AKGATHQNYIEFDDRAEGIKHAIDIAEPGDTVVLASKGREPYQIMPGHIKVPHRDDLIGLEAAYKKFGGG
PVDQ

>SAEMRSA109190 conserved hypothetical protein
MKQLHPNEVGVYALGGLGEIGKNTYAVEYKDEIVIIDAGIKFPDDNLLGIDYVIPDYTYLVQNQDKIVGL
FITHGHEDHIGGVPFLLKQLNIPIYGGPLALGLIRNKLEEHHLLRTAKLNEINEDSVIKSKHFTISFYLT
THSIPETYGVIVDTPEGKVVHTGDFKFDFTPVGKPANIAKMAQLGEEGVLCLLSDSTNSLVPDFTLSERE
VGQNVDKIFRNCKGRIIFATFASNIYRVQQAVEAAIKNNRKIVTFGRSMENNIKIGMELGYIKAPPETFI
EPNKINTVPKHELLILCTGSQGEPMAALSRIANGTHKQIKIIPEDTVVFSSSPIPGNTKSINRTINSLYK
AGADVIHSKISNIHTSGHGSQGDQQLMLRLIKPKYFLPIHGEYRMLKAHGETGVECGVEEDNVFIFDIGD
VLALTHDSARKAGRIPSGNVLVDGSGIGDIGNVVIRDRKLLSEEGLVIVVVSIDFNTNKLLSGPDIISRG
FVYMRESGQLIYDAQRKIKTDVISKLNQNKDIQWHQIKSSIIETLQPYLFEKTARKPMILPVIMKVNEQK
ESNNK

>SaurJH9 1173 cell cycle protein

MTEYMKNFRSILRYIGKTSKFIDYPLLVTYIVLSLIGLVMVYSASMVPATKGTLTGGIDVPGTYFYNRQLAYVIMSFIIVFFIAFLMNVKLLSNIKVQKGM
IITIVSLLLLTLVIGKDINGSKSWINLGFMNLQASELLKIAIILYIPFMISKKMPRVLSKPKLILSPIVLALGCTFLVFLQKDVGQTLLILIILVAIIFYSGIGVNKV
LRFGIPAVLGFLVVFVIALMAGWLPSYLTARFSTLTDPFQFESGTGYHISNSLLAIGNGGVFGKGLGNSAMKLGYLPEPHTDFIFAIICEELGLIGGLLVI
TLEFFIVYRAFQFANKTSSYFYKLVCVGIATYFGSQTFVNIGGISATIPLTGVPLPFISFGGSSMISLSIAMGLLLIVGKQIKVDQQRKKQQQKVDIRRQF
N

>SAEMRSA109800 putative glutamate racemase
MNKPIGVIDSGVGGLTVAKEIMRQLPNETIYYLGDIGRCPYGPRPGEQVKQYTVEIARKLMEFDIKMLVIACNTATAVALEYLQKTLSIPVIGVIEPGA
RTAIMTTRNQNVLVLGTEGTIKSEAYRTHIKRINPHVEVHGVACPGFVPLVEQMRYSDPTITSIVIHQTLKRWRNSESDTVILGCTHYPLLYKLIYDYFG
GKKTVISSGLETAREVSALLTFSNEHASYTEHPDHRFFATGDPTHITNIIKEWLNLSVNVERISVND

>SAEMRSA110140 penicillin-binding protein 1
MAKQKIKIKKNKIGAVLLVGLFGLLFFILVLRISYIMITGHSNGQDLVMKANEKYLVKNAQQPERGKIYDRNGKVLAEDVERYKLVAVIDKKASANSKK
PRHVVDKKETAKKLSTVIDMKPEEIEKRLSQKKAFQIEFGRKGTNLTYQDKLKIEKMNLPGISLLPETERFYPNGNFASHLIGRAQKNPDTGELKGALG
VEKIFDSYLSGSKGSLRYIHDIWGYIAPNTKKEKQPKRGDDVHLTIDSNIQVFVEEALDGMVERYQPKDLFAVVMDAKTGEILAYSQRPTFNPETGKD
FGKKWANDLYQNTYEPGSTFKSYGLAAAIQEGAFDPDKKYKSGHRDIMGSRISDWNRVGWGEIPMSLGFTYSSNTLMMHLQDLVGADKMKSW
YERFGFGKSTKGMFDGEAPGQIGWSNELQQKTSSFGQSTTVTPVQMLQAQSAFFNDGNMLKPWFVNSVENPVSKRQFYKGQKQIAGKPITKDT
AEKVEKQLDLVVNSKKSHAANYRIDGYEVEGKTGTAQVAAPNGGGYVKGPNPYFVSFMGDAPKKNPKVIVYAGMSLAQKNDQEAYELGVSKAFK
PIMENTLKYLNVGKSKDDTSNAEYSKVPDVEGQDKQKAIDNVSAKSLEPVTIGTGTQIKAQSIKAGNKVLPHSKVLLLTDGDLTMPDMTGWTKEDV
IAFENLTNIKVNLKGSGFVSHQSISKGQKLTEKDKIDVEFSSENVDSNSTNNSDSNSDDKKKSDSKTDKDKSD

>SAEMRSA110150 phospho-N-acetylmuramoyl-pentapeptide-transfera se
MIFVYALLALVITFVLVPVLIPTLKRMKFGQSIREEGPQSHMKKTGTPTMGGLTFLLSIVITSLVAIIFVDQANPIILLLFVTIGFGLIGFIDDYIIVVKKNNQ
GLTSKQKFLAQIGIAIIFFVLSNVFHLVNFSTSIHIPFTNVAIPLSFAYVIFIVFWQVGFSNAVNLTDGLDGLATGLSIIGFTMYAIMSFVLGETAIGIFCIIM
LFALLGFLPYNINPAKVFMGDTGSLALGGIFATISIMLNQELSLIFIGLVFVIETLSVMLQVASFKLTGKRIFKMSPIHHHFELIGWSEWKVVTVFWAVG
LISGLIGLWIGVH

>SAEMRSA110160 UDP-N-acetylmuramoylalanine--D-glutamate ligase
MLNYTGLENKNVLVVGLAKSGYEAAKLLSKLGANVTVNDGKDLSQDAHAKDLESMGISVVSGSHPLTLLDNNPIIVKNPGIPYTVSIIDEAVKRGLKIL
TEVELSYLISEAPIIAVTGTNGKTTVTSLIGDMFKKSRLTGRLSGNIGYVASKVAQEVKPTDYLVTELSSFQLLGIEKYKPHIAIITNIYSAHLDYHENLENY
QNAKKQIYKNQTEEDYLICNYHQRQVIESEELKAKTLYFSTQQEVDGIYIKDSFIVYKGVRIINTEDLVLPGEHNLENILAAVLACILAGVPIKAIIDSLTTF
SGIEHRLQYVGTNRTNKYYNDSKATNTLATQFALNSFNQPIIWLCGGLDRGNEFDELIPYMENVRAMVVFGQTKAKFAKLGNSQGKSVIEANNVE
DAVDKVQDIIEPNDVVLLSPACASWDQYSTFEERGEKFIERFRAHLPSY

>SAEMRSA110170 putative cell division protein
LMDDKTKNDQQESNEDKDELELFTRNTSKKRRQRKRSKATHFSNQNKDDTSQQTDFDEEIYLINKDFKKE
ESNDENNDSASSHANNNNIDDSTDSNIENEDYRYNQEIDDQNESNGIAVDNEQPQSAPKEQNSDSNDEET
VTKKERKSKVTQLKPLTLEEKRKLRRKRQKRIQYSVITILVLLIAVILIYMFSPLSKIAHVNINGNNHVS
TSKINKVLGVKNDSRMYTFSKKNAINDLEEDPLIKSVEIHKQLPNTLNVDITENEIIALVKYKGKYLPLL
ENGKLLKGSNDVKINDAPVMDGFKGTKEDDMIKALSEMTPEVRRYIAEVTYAPSKNKQSRIELFTTDGLQ
VIGDISTISKKMKYYPQMSQSLSRDSSGKLKTRGYIDLSVGASFIPYRGNTSSQSESDKNVTKSSQEENQ
AKEELQSVLNKINKQSSKNN

>AA076 0589cell division protein FtsA
MEEHYYVSIDIGSSSVKTIVGEKFHNGINVIGTGQTYTSGIKNGLIDDFDIARQAIKDTIKKASIASGVD
IKEVFLKLPIIGTEVYDESNEIDFYEDTEINGSHIEKVLEGIREKNDVQETEVINVFPIRFIVDKENEVS
DPKELIARHSLKVEAGVIAIQKSILINMIKCVEACGVDVLDVYSDAYNYGSILTATEKELGACVIDIGED
VTQVAFYERGELVDADSIEMAGRDITDDIAQGLNTSYETAEKVKHQYGHAFYDSASDQDIFTVEQVDSDE
TVQYTQKDLSDFIEARVEEIFFEVFDVLQDLGLTKVNGGFIVTGGSANLLGVKELLSDMVSEKVRIHTPS
QMGIRKPEFSSAISTISSSIAFDELLDYVTINYHDNEETEEDVIDVKDKDNESKLGGFDWFKRKTNKKDT
HENEVESTDEEIYQSEDNHQEHKQNHEHVQDKDKDKEESKFKKLMKSLFE

>SAEMRSA110190 cell division protein FtsZ
MLEFEQGFNHLATLKVIGVGGGGNNAVNRMIDHGMNNVEFIAINTDGQALNLSKAESKIQIGEKLTRGLG

AGANPEIGKKAAEESREQIEDAIQGADMVFVTSGMGGGTGTGAAPVVAKIAKEMGALTVGVVTRPFSFEG
RKRQTQAAAGVEAMKAAVDTLIVIPNDRLLDIVDKSTPMMEAFKEADNVLRQGVQGISDLIAVSGEVNLD
FADVKTIMSNQGSALMGIGVSSGENRAVEAAKKAISSPLLETSIVGAQGVLMNITGGESLSLFEAQEAAD
IVQDAADEDVNMIFGTVINPELQDEIVVTVIATGFDDKPTSHGRKSGSTGFGTSVNTSSNATSKDESFTS
NSSNAQATDSVSERTHTTKEDDIPSFIRNREERRSRRTRR

>SAEMRSA110290 lipoprotein signal peptidase
MHKKYFIGTSILIAVFVVIFDQVTKYIIATTMKIGDSFEVIPHFLNITSHRNNGAAWGILSGKMTFFFII
TIIILIALVYFFIKDAQYNLFMQVAISLLFAGALGNFIDRILTGEVVDFIDTNIFGYDFPIFNIADSSLT
IGVILIIIALLKDTSNKKEKEVK

>SAEMRSA110540 conserved hypothetical protein
LKTGRIVKSISGVYQVDVNGERFNTKPRGLFRKKKFSPVVGDIVEFDVQNINEGYIHQVYERKNELKRPP
VSNIDTLVIVMSAVEPNFSTQLLDRFLVIAHSYQLDARVLVTKKDKTPIEKQLEINELLKIYENIGYETE
FIGNDDDRKKIVEAWPAGLIVLSGQSGVGKSTFLNHYRPELNLETNDISKSLNRGKHTTRHVELFERQNG
YIADTPGFSALDFDHIDKDEIKDYFLELNRYGETCKFRNCNHIKEPNCNVKHQLEIGNIAQFRYDHYLQL
FNEISNRKVRY

>SAEMRSA110620 putative fatty acid synthesis protein
MVKLAIDMMGGDNAPDIVLEAVQKAVEDFKDLEIILFGDEKKYNLNHERIEFRHCSEKIEMEDEPVRAIK
RKKDSSMVKMAEAVKSGEADGCVSAGNTGALMSAGLFIVGRIKGVARPALVVTLPTIDGKGFVFLDVGAN
ADAKPEHLLQYAQLGDIYAQKIRGIDNPKISLLNIGTEPAKGNSLTKKSYELLNHDHSLNFVGNIEAKTL
MDGDTDVVVTDGYTGNMVLKNLEGTAKSIGKMLKDTIMSSTKNKLAGAILKKDLAEFAKKMDYSEYGGSV
LLGLEGTVVKAHGSSNAKAFYSAIRQAKIAGEQNIVQTMKETVGESNE

>SAI7S6 1009020 UMP kinase
VSSLLVYVTYIHDREDKKMAQISKYKRVVLKLSGEALAGEKGFGINPVIIKSVAEQVAEVAKMDCEIAVI
VGGGNIWRGKTGSDLGMDRGTADYMGMLATVMNALALQDSLEQLDCDTRVLTSIEMKQVAEPYIRRRAIR
HLEKKRVVIFAAGIGNPYFSTDTTAALRAAEVEADVILMGKNNVDGVYSADPKVNKDAVKYEHLTHIQML
QEGLQVMDSTASSFCMDNNIPLTVFSIMEEGNIKRAVMGEKIGTLITK

>SAEMRSA110920 ribosome recycling factor
MSDIINETKSRMQKSIESLSRELANISAGRANSNLLNGVTVDYYGAPTPVQQLASINVPEARLLVISPYD
KTSVADIEKAIIAANLGVNPTSDGEVIRIAVPALTEERRKERVKDVKKIGEEAKVSVRNIRRDMNDQLKK
DEKNGDITEDELRSGTEDVQEATDNSIKEIDQMIADKEKDIMSV

>SAEMRSA110970 DNA polymerase III PolC-type
LAMTEQQKFKVLADQIKISNQLDAEILNSGELTRIDVSNKNRTWEFHITLPQFLAHEDYLLFINAIEQEF
KDIANVTCRFTVTNGTNQDEHVIKYFGHCIDQTALSPKVKGQLKQKKLIMSGKVLKVMVSNDIERNHFDK
ACNGSLIKAFRNCGFDIDKIIFETNDNDQEQNLASLEAHIQEEDEQSARLATEKLEKMKAEKAKQQDNNE
SAVDKCQIGKPIQIENIKPIESIIEEEYKVAIEGVIFDINLKELKSGRHIVEIKVTDYTDSLVLKMFTRK
NKDDLEHFKALSVGKWVRAQGRIEEDTFIRDLVMMMSDIEEIKKATKKDKAEEKRVEFHLHTAMSQMDGI
PNISAYVKQAADWGHPAIAVTDHNVVQAFPDAHAAAEKHGIKMIYGMEGMLVDDGVPIAYKPQDVVLKDA
TYVVFDVETTGLSNQYDKIIELAAVKVHNGEIIDKFERFSNPHERLSETIINLTHITDDMLVDAPEIEEV
LTEFKEWVGDAIFVAHNASFDMGFIDTGYERLGFGPSTNGVIDTLELSRTINTEYGKHGLNFLAKKYGVE
LTQHHRAIYDTEATAYIFIKMVQQMKELGVLNHNEINKKLSNEDAYKRARPSHVTLIVQNQQGLKNLFKI
VSASLVKYFYRTPRIPRSLLDEYREGLLVGTACDEGELFTAVMQKDQSQVEKIAKYYDFIEIQPPALYQD
LIDRELIRDTETLHEIYQRLIHAGDTAGIPVIATGNAHYLFEHDGIARKILIASQPGNPLNRSTLPEAHF
RTTDEMLNEFHFLGEEKAHEIVVKNTNELADRIERVVPIKDELYTPRMEGANEEIRELSYANARKLYGED
LPQIVIDRLEKELKSIIGNGFAVIYLISQRLVKKSLDDGYLVGSRGSVGSSFVATMTEITEVNPLPPHYI
CPNCKRSEFFNDGSVGSGFDLPDKTCETCGAPLIKEGQDIPFETFLGFKGDKVPDIDLNFSGEYQPNAHN
YTKVLFGEDKVFRAGTIGTVAEKTAFGYVKGYLNDQGIHKRGAEIDRLVKGCTGVKRTTGQHPGGIIVVP
DYMDIYDFTPIQYPADDQNSAWMTTHFDFHSIHDNVLKLDILGHDDPTMIRMLQDLSGIDPKTIPVDDKE
VMQIFSTPESLGVTENEILCKTGTFGVPEFGTGFVRQMLEDTKPTTFSELVQISGLSHGTDVWLGNAQEL

IKTGICDLSSVIGCRDDIMVYLMYAGLEPSMAFKIMESVRKGKGLTEEMIETMKENEVPDWYLDSCLKIK
YMFPKAHAAAYVLMAVRIAYFKVHHPLYYYASYFTIRASDFDLITMIKDKTSIRNTVKDMYSRYMDLGKK
EKDVLTVLEIMNEMAHRGYRMQPISLEKSQAFEFIIEGDTLIPPFISVPGLGENVAKRIVEARDDGPFLS
KEDLNKKAGLSQKIIEYLDELGSLPNLPDKAQLSIFDM

>SAEMRSA111050 putative riboflavin biosynthesis protein
MKVIEVTHPIQSKQYITEDVAMAFGFFDGMHKGHDKVFDILNEIAEARSLKKAVMTFDPHPSVVLNPKRK
RTTYLTPLSDKIEKISQHDIDYCIVVNFSSRFANVSVEDFVENYIIKNNVKEVIAGFDFTFGKFGKGNMT
VLQEYDAFNTTIVSKQEIENEKISTTSIRQDLINGELQKANDALGYIYSIKGTVVQGEKRGRTIGFPTAN
IQPSDDYLLPRKGVYAVSIEIGTENKLYRGVANIGVKPTFHDPNKAEVVIEVNIFDFEDNIYGERVTVNW
HHFLRPEIKFDGIDPLVKQMNDDKSRAKYLLAVDFGDEVAYNI

>SAI7S6 1009170 DNA translocase FtsK
VRQLAQAKKKSTAKKKTASKKRTNSRKKKNDNPIRYVIAILVVVLMVLGVFQLGIIGRLIDSFFNYLFGY
SRYLTYILVLLATGFITYSKRIPKTRRTAGSIVLQIALLFVSQLVFHFNSGIKAEREPVLSYVYQSYQHS
HFPNFGGGVLGFYLLELSVPLISLFGVCIITILLLCSSVILLTNHQHRDVAKVALENIKAWFGSFNEKMS
ERNQEKQLKREEKARLKEEQKARQNEQPQIKDVSDFTEVPQERDIPIYGHTENESKSQCQPSRKKRVFDA
ENSSNNIVNHQADQQEQLTEQTHNSVESENTIEEAGEVTNVSYVVPPLTLLNQPAKQKATSKAEVQRKGQ
VLENTLKDFGVNAKVTQIKIGPAVTQYEIQPAQGVKVSKIVNLHNDIALALAAKDVRIEAPIPGRSAVGI
EVPNEKISLVSLKEVLDEKFPSNNKLEVGLGRDISGDPITVPLNEMPHLLVAGSTGSGKSVCINGIITSI
LLNAKPHEVKLMLIDPKMVELNVYNGIPHLLIPVVTNPHKAAQALEKIVAEMERRYDLFQHSSTRNIKGY
NELIRKQNQELDEKQPELPYIVVIVDELADLMMVAGKEVENAIQRITQMARAAGIHLIVATQRPSVDVIT
GIIKNNIPSRIAFAVSSQTDSRTIIGTGGAEKLLGKGDMLYVGNGDSSQTRIQGAFLSDQEVQDVVNYVV
EQQQANYVKEIEPDAPVDKSEMKSEDALYDEAYLFVVEQQKASTSLLQRQFRIGYNRASRLMDDLERNQV
IGPQKGSKPRQVLIDLNNDEV

>SAEMRSA112010 putative membrane protein
MMIIVMLLLSYLIGAFPSGFVIGKLFFKKDIRQFGSGNTGATNSFRVLGRPAGFLVTFLDIFKGFITVFF
PLWLPVHADGPISTFFTNGLIVGLCAILGHVYPVYLKFQGGKAVATSAGVVLGVNPILLLILAIIFFVVL
KIFKYVSLASIVAAICCVIGSLIIQDYILLVVSFLVSIILIIRHRSNIARIFRGEEPKIKWM

>SAEMRSA112200 tryptophan synthase beta chain
MNKQIQTEADELGFFGEYGGQYVPETLMPAIIELKKAYKEAKADPEFQRELEYYLSEYVGRATPLTYAAS
YTESLGGAKIYLKREDLNHTGAHKINNALGQALLAKRMGKKKLVAETGAGQHGVASATVAALFDMELVVF
MGSEDIKRQQLNVFRMELLGAKVVAVEDGQGTLSDAVNKALQYWVSHVDDTHYLLGSALGPDPFPTIVRD
FQSVIGKEIKSQILKKEGRLPDAIVACIGGGSNAIGTFYPFIKDDVALYGVEAAGQGEDTDKHALAIGKG
SPGVLHGTKMYLIQDEGGQVQLAHSISAGLDYPGIGPEHSYYHDIGRVTFENASDTQAMNALINFTKHEG
IIPAIESAHALSYVERLAPTMSKEDIIVVTISGRGDKDMETIRQYMAERGLAND

>SAEMRSA112560 aspartokinase II
MVTRSVLKFGGSSVSDFTKIKRIAEMLKERVNQDEQLIVVVSAMGNTTDQLMTNVSTLTKAPKQQELALL
LTTGEQQTVSYLSMVLNDIGVNAKAMTGYQAGIKTIGHHLKSKIAQINPQTFEQAFQENDILVVAGFQGI
NEHQELTTLGRGGSDTTAVALAVSNQIPCEIYTDVDGVYATDPRLLPKAKRLDIVSYEEMMEMSALGAGV
LETRSVELAKNYNIPLYLGKTLSNVKGTWIMSNEEILEKKAVTGVALDKHMMHVTISYPLPDNQLLTQLF
TELEEGAVNVDMISQIVNLDGLQLSFTIKDSDFHQISMILETLKNQYEALAYKINEHYVKISLIGSGMRD
MSGVASKAFLTLIENNIPFYQTTTSEISISYVIDDFNGQQAVEKLYDAFNI

>SAEMRSA112570 aspartate semialdehyde dehydrogenase
MTKLAVVGATGLVGTKMLETLNRKNIPFDELVLFSSARSAGQEVEFQEKTYTVQELTDARASEHFDYVLM
SAGGGTSEHFAPLFEKAGAIVIDNSSQWRMAEDIDLIVPEVNEPTFTRGIIANPNCSTIQSVVPLKVLQD
AYGLKRVAYTTYQAVSGSGMKGKKDLAEGVNGKAPEAYPHPIYNNVLPHIDVFLENGYTKEEQKMIDETR
KILNAPDLKVTATCARVPVQDSHSVEIDVTLDKEATAEDIKALFDQDDRVVLVDNPENNEYPMAINSTNK
DEVFVGRIRRDDSLENTFHVWCTSDNLLKGAALNAVQVLEQVMRLKGAN

>SAEMRSA112590 dihydrodipicolinate reductase
VKILLIGYGAMNQRVARLAEEKGHEIVGVIENTPKATTPYQQYQHIADVKDADVAIDFSNPNLLFPLLDE
EFHLPLVVATTGEKEKLLNKLDELSQNMPVFFSANMSYGVYALTKILAAAVPLLDDFDIELTEAHHNKKV
DAPSGTLEKLYDVIVSLKENVTPVYDRHELNEKRQPQDIGIHSIRGGTIVGEHEVLFAGTDETIQITHRA
QSKDIFANGAIQAAERLVNKPNGLYTFDNL

>SAEMRSA112600 putative tetrahydrodipicolinate acetyltransferase
MVQHLTAEEIIQYISDAKKSTPIKVYLNGNFEGITYPESFKVFGSEQSKVIFCEADDWKPFYEAYGSQFE
DIEIEMDRRNSAIPLKDLTNTNARIEPGAFIREQAIIEDGAVVMMGATINIGAVVGEGTMIDMNATLGGR
ATTGKNVHVGAGAVLAGVIEPPSASPVIIEDDVLIGANAVILEGVRVGKGAIVAAGAIVTQDVPAGAVVA
GTPAKVIKQASEVQDTKKEIVAALRKLND

>SAEMRSA112810 putative UDP-N-acetylglucosamine-N-acetylmuramyl-(pentapeptide)pyr ophosphoryl-undecaprenol N-acetylglucosamine transferase
MKKIAFTGGGTVGHVSVNLSLIPTALSQGYEALYIGSKNGIEREMIESQLPEIKYYPISSGKLRRYISLE
NAKDVFKVLKGILDARKVLKKEKPDLLFSKGGFVSVPVVIAAKSLNIPTIIHESDLTPGLANKIALKFAK
KIYTTFEETLNYLPKEKADFIGATIREDLKNGNAHNGYQLTGFNENKKVLLVMGGSLGSKKLNSIIRENL
DALLQQYQVIHLTGKGLKDAQVKKSGYIQYEFVKEELTDLLAITDTVISRAGSNAIYEFLTLRIPMLLVP
LGLDQSRGDQIDNANHFADKGYAKAIDEEQLTAQILLQELNEMEQERTRIINNMKSYEQSYTKEALFDKM
IKDALN

>SAEMRSA113110 penicillin-binding protein 2
MTENKGSSQPKKNGNNGGKSNSKKNRNVKRTIIKIIGFMIIAFFVVLLLGILLFAYYAWKAPAFTEAKLQ
DPIPAKIYDKNGELVKTLDNGQRHEHVNLKDVPKSMKDAVLATEDNRFYEHGALDYKRLFGAIGKNLTGG
FGSEGASTLTQQVVKDAFLSQHKSIGRKAQEAYLSYRLEQEYSKDDIFQVYLNKIYYSDGVTGIKAAAKY
YFNKDLKDLNLAEEAYLAGLPQVPNNYNIYDHPKAAEDRKNTVLYLMHYHKRITDKQWEDAKKIDLKANL
VNRTAEERQNIDTNQDSEYNSYVNFVKSELMNNKAFKDENLGNVLQSGIKIYTNMDKDVQKTLQNDVDNG
SFYKNKDQQVGATILDSKTGGLVAISGGRDFKDVVNRNQATDPHPTGSSLKPFLAYGPAIENMKWATNHA
IQDESSYQVDGSTFRNYDVKSHGTVSIYDALRQSFNIPALKAWQSVKQNAGNDAPKKFAAKLGLNYEGDI
GPSEVLGGSASEFSPTQLASAFAAIANGGTYNNAHSIQKVVTRDGETIEYDHTSHKAMSDYTAYMLAEML
KGTFKPYGSAYGHGVSGVNMGAKTGTGTYGAETYSQYNLPDNAAKDVWINGFTPQYTMSVWMGFSKVKQY
GENSFVGHSQQEYPQFLYENVMSKISSRDGEDFKRPSSVSGSIPSINVSGSQDNNTTNRSAHGGSDTSAN
SSGTAQSNNNTRSQQSRNSGGLTGIFN

>SAEMRSA113140 conserved hypothetical protein
MDKYQLKARPVVIRRELLDHYSDLGLDEQDLVILLKLIYASETSNKQPSIELLQKGSTMQPRDITMVIQN
LIQRELLELQVQKDEEGRFTEYMNLDPFFEKLSHILKQQSMETKEQNSKEKFKQLFRVLEDTFARPLSPY
EIETLNQWIDVDKHDTAIIQAALDEANSLNKLSFKYMDRILLNWKKNNVKTIDDSRKIREKFNKPKMTHT
VKTVPKFDWLNGENLDGK

>SAEMRSA113270 chorismate synthase
MRYLTSGESHGPQLTVIVEGIPANLEIKVEDINKEMFKRQGGYGRGRRMQIEKDTVEIVSGVRNGYTLGS
PITMVVTNDDFTHWRKIMGAAPISDEERENMKRTITKPRPGHADLVGGMKYNHRDLRNVLERSSARETAA
RVAVGALCKVLLEQLDIEIYSRVVEIGGIKDKDFYDSETFKANLDRNDVRVIDDGIAQAMRDKIDEAKND
GDSIGGVVQVVVENMPVGVGSYVHYDRKLDGRIAQGVVSINAFKGVSFGEGFKAAEKPGSEIQDEILYNT
ELGYYRGSNHLGGLEGGMSNGMPIIVNGVMKPIPTLYKPLNSVDINTKEDFKATIERSDSCAVPAASIVC
EHVVAFEIAKALLEEFQSNHIEQLKQQIIERRQLNIEF

>SAEMRSA113370 cytidylate kinase
MKAINIALDGPAAAGKSTIAKRVASELSMIYVDTGAMYRALTYKYLKLNKTEDFAKLVDQTTLDLTYKAD
KGQCVILDNEDVTDFLRNNDVTQHVSYVASKEPVRSFAVKKQKELAAEKGIVMDGRDIGTVVLPDADLKV
YMIASVEERAERRYKDNQLRGIESNFEDLKRDIEARDQYDMNREISPLRKADDAVTLDTTGKSIEEVTDE
ILAMVSKIK

>SAEMRSA114480 biotin carboxyl carrier protein of acetyl-CoA carboxylase
MNFKEIKELIEILDKSTLTEINIEDTKGKVTLKKEKETEIITPQISQMPVEAAAMPMPQVQSTDSNKTEA
PKPTSDNHKTINAPMVGTFYKSPSPDEEAYVQVGDTVSNETTVCILEAMKLFNEIQAEISGEIVEILVED
GQMVEYGQPLFKVK

>SAEMRSA114490 putative elongation factor P
MISVNDFKTGLTISVDNAIWKVIDFQHVKPGKGSAFVRSKLRNLRTGAIQEKTFRAGEKVEPAMIENRRM
QYLYADGDNHVFMDNESFEQTELSSDYLKEELNYLKEGMEVQIQTYEGETIGVELPKTVELTVTETEPGI
KGDTATGATKSATVETGYTLNVPLFVNEGDVLIINTGDGSYISRG

>SAEMRSA114580 putative shikimate kinase
MNHDKSPIILIGFMGTGKSTIGKYVADEQNLSFIDIDSYIEEKYKLTIPEIFSKHGEQYFRNLEFTCLQE
CINTADIIATGGGIIESEEAFNFLKNQKNIIWLDCNIDIIYSRINDDPHRPNANNKTIMQLNDLYCSRIL
RYNEIAFKKFDSHLLSISEIYYELLNLIKASDQY

>SAEMRSA114820 DNA primase
LRIDQSIINEIKDKTDILDLVSEYVKLEKRGRNYIGLCPFHDEKTPSFTVSEDKQICHCFGCKKGGNVFQ
FTQEIKDISFVEAVKELGDRVNVAVDIEATQSNSNVQIASDDLQMIEMHELIQEFYYYALTKTVEGEQAL
TYLQERGFTDALIKERGIGFAPDSSHFCHDFLQKKGYDIELAYEAGLLSRNEENFSYYDRFRNRIMFPLK
NAQGRIVGYSGRTYTGQEPKYLNSPETPIFQKRKLLYNLDKARKSIRKLDEIVLLEGFMDVIKSDTAGLK
NVVATMGTQLSDEHITFIRKLTSNITLMFDGDFAGSEATLKTGQHLLQQGLNVFVIQLPSGMDPDEYIGK
YGNDAFTTFVKNDKKSFAHYKVSILKDEIAHNDLSYERYLKELSHDISLMKSSILQQKAINDVAPFFNVS
PEQLANEIQFNQAPANYYPEDEYGGYDEYGGYIEPEPIGMAQFDNLSRQEKAERAFLKHLMRDKDTFLNY
YESVDKDNFTNQHFKYVFEVLHDFYAENDQYNISDAVQYVNSNELRETLISLEQYNLNDEPYENEIDDYV
NVINEKGQETIESLNHKLREATRIGDVELQKYYLQQIVAKNKERM

>SAEMRSA115060 conserved hypothetical protein
MSDNIVAIYGDVPELVEKQSAEIISQFLKSDRDDFNFVKYNLYETEIAPIVEETLTLPFFSDKKAILVKN
AYIFTGEKAPKDMAHNVDQLIEFIEKYDGENLIVFEIYQNKLDERKKLTKTLKKHARLKKIEQMSEEEIK
KWIQSKLNENFKDIKRDALDLFIELTGINFNIVSQEIEKLILFLGDRPTINKQDVNQIINRSLEQNVFLL
TEYIQKRKKEQAIHLVKDLITMKEEPIKLLALITSNYRLFYQCKILSQKGYSGQQIAKTIGVHPYRVKLA
LGQVRHYQLDELLNIIDACAETDYKLKSSYMDKQLILELFILSL

>SAEMRSA115130 conserved hypothetical protein
MKKIVLYGGQFNPIHTAHMIVASEVFHELQPDEFYFLPSFMSPLKKHNNFIDVQHRLTMIQMIIDELGFG
DICDDEIKRGGQSYTYDTIKAFKEQHKDSELYFVIGTDQYNQLEKWYQIEYLKEMVTFVVVNRDKNSQNV
ENAMIAIQIPRVDISSTMIRQRVSEGKSIQVLVPKSVENYIKGEGLYEH

>SAEMRSA115170 conserved hypothetical protein
MGLVRKFFMPNSYVQSIFQIDLDKLVDKGVKGIITDLDNTLVGWDVKEPTERVKAWFKEANEKGITITIV
SNNNESRVASFSQHLDIDFIFKARKPMGKAFDKAITKMNIRPDQTVVIGDQMLTDVFGGNRRGLYTIMVV
PVKRTDGFITKFNRLIERRLLRHFSKKGYITWEEN

>SAEMRSA115280 transcription elongation factor
MENQKQYPMTQEGFEKLERELEELKTVKRPEVVEKIKVARSFGDLSENSEYDAAKDEQGFIEQDIQRIEH
MLRNALIIEDTGDNNVVKIGKTVTFVELPGDEEESYQIVGSAESDAFNGKISNESPMAKALIGKGLDDEV
RVPLPNGGEMNVKIVNIQ

>SAEMRSA115540 putative protein-export membrane protein
VKKSSRIIAFLLLVVLLFAGMAATYKSVIKNVNLGLDLQGGFEVLYQVDPLNKGDKIDKKALQSTAQTLE
NRVNVLGVSEPKIQVEEPNRIRVQLAGVTDQNEARKILSSQANLTIRDAEDKVKLSGSDIKQGSAKQEFK
QETNQPTVTFKVKDKNKFKKVTEEISKKRDNVMVVWLDFKKGDSYKKEAQKKNPKFISAASVDQPINSDS
VEISGGFKGQEGVKKAKQIAELLNAGSLPVDLKEIYSNSVGAQFGQDALDKTVFASFIGVALIYLFMLGF
YRLPGLVAIIALTTYIYLTLVAFNFISGVLTLPGLAALVLGVGMAVDANIIMYERIKDELRIGRTIKQAF

SKANKSSFLTIFDSNLTTVIAAAVLFFFGESSVKGFATMLLLGILMIFVTAVFLSRFLLSLLVSSNIFKN
QFWLFGVKKNKRHDINEGVDVHDLKTSFEKWNFVKLAKPLIGVSILIVVVGLVILYIFKLNLGIDFSSGT
RVDFQSKQAITQQKVEQVVKDSGLKADQIQINGKDNKVATVQFKDDLTRAQDNKLSDNIKSKFGDTPQIN
TVSPIIGQELAKNAMLALIYASIGIIIYVSLRFEWRMGLSSVLALLHDVFIIVAIFSLFRIEVDLTFIAA
VLTIVGYSINDTIVTFDRVRENLQKVKVITTTEQIDDIVNRSIRQTMTRSINTVLTVIVVVVAILFFGAP
TIFNFTLALFIGLISGVFSSIFIAVPLWGIMKKRQLKKSPKHKLVVYKEKKSNDEKILV

>SAEMRSA115590 Holliday junction DNA helicase RuvA
MYAYVKGKLTHLYPTHVVVETAGVGYEIQTPNSYRFQKQLDHEVLIHTSLIVREDAQLLYGFSSEEEKDM
FLSLIKVTGIGPKSALAILATSTPNEVKRAIENENDTYLTKFPGIGKKTARQIVLDLKGKVKITEEDSDS
LLQVDATSTVQDQFVQEAMLALEALGYSKRELAKVEKTLNKNKYDSVDEAVKAGLQLVVS

>SaurJH9 1738 bacterial translation initiation factor 3 (bIF-3)
VYKEERVFYTCSSFLLGANFVKILEVSTIAKDQTQINDKIRAKELRLIGQDGEQIGVKSKREALEMAERV
DLDLVVVAPNAKPPVARIMDYGKFKFEQQKKEKEMKKKQKIINVKEIRLSPTIEEHDFQTKLKNGRKFLT
KGDKCKVSIRFRGRAITHKEIGQRVLEKYADECKDIATVEQKPKMDGRQMFIMLAPTAEK

>SAEMRSA115950 chromosome replication initiation/membrane attachment protein
MGRQAFEFGLRPKDQFKVMQHFDLNTNHLEVLNRLYTPLIGTQAVGLYHFMTQFVKESHNETLILSHYIF
MNELKINLLEFRQQMDLLEAIGLLKAFVKHDEQETQFVYQLIQPPSAHLFFNDPMLSIFLYSEVEHRRFH
ELKKYFEYQQIDLSEFKQVTRQFTDVFKVPSTKIDIDTSDIPINEPYQGIDLSNESFDFEMLRQMLGKHF
ISQDIVTKDAKRLITQLATLYGLTADGMKHVILNSITSGQQLSFEEMRKQARSYYLMEHENQMPKLQVKS
PVTSSSTGKSSEVNPKPQSDEWFELLEQTSPIDMLASWSESEPTISQKTMVEELIEREKMSFGVINILLQ
FVMLKEDMKLPKAYILEIASNWKKKGIKTAKEAYNYAKKVNQPKNEGSSGNYQKRGSYYGQRNRISKEKT
PKWLENRDKPSEEDSAKDNSVDDQQLEQDRQAFLDKLSKKWEEDSQ

>SAEMRSA116120 DNA polymerase III alpha subunit
MVAYLNIHTAYDLLNSSLKIEDAVRLAVSENVDALAITDTNVLYGFPKFYDTCIANNIKPIFGMTIYVTN
GLNTVETVVLAKNNDGLKDLYQLSSEIKMNALEHVSFELLKRFSNNMIIIFKNVADEHRDIVQVFDSHED
TYLDHRSVLVQGIKHVWIQNVCYQTRQDADTISALAAIRDNTKLDLIHDQEDFGAHFLTENEIQQLDVNQ
EYLTQVDVIAQKCNAELKYHQSLLPQYQTPNDESAKKYLWRVLVTQLKKLELNYDVYLERLKYEYKVITN
MGFEDYFLIVSDLIHYAKTHDVMVGPGRGSSAGSLVSYLLGITTIDPIKFNLLFERFLNPERVTMPDIDI
DFEDTRRERVIQYVQEKYGELHVSGIVTFGHLLARAVARDVGRIMGFDEVTLNEISSLIPHKLGITLDEA
YQIDDFKKFVHRNHRHERWFSICKKLEGLPRHTSTHAAGIIINDHPLYEYAPLTKGDTGLLTQWTMTEAE
RIGLLKIDFLGLRNLSIIHQILTQVKKDLGINIDIENIPFDDQKVFELLSQGDTTGIFQLESDGVRSVLK
KLKPEHFEDIVAVTSLYRPGPMEEIPTYITRRHDPSKVQYLHPHLEPILKNTYGVIIYQEQIMQIASTFA
NFSYGEADILRRAMSKKNRAVLESERQHFIEGAKQNGYHEDISKQIFDLILKFADYGFPRAHAVSYSKIA
YIMSYLKVRYPNYFYANILSNVIGSEKKTAQMIEEAKKQGITILPPNINESHCFYKPSQEGIYLSIGTIK
GVGYQSVKVIVDERYQNGKFKDFFDFARRIPKRVKTRKLLEALILVGAFDAFGKTRSTLLQAIDQVLDGD
LNIEQDGFLFDILTPKQMYEDKEELPDALISQYEKEYLGFYVSQHPVDKKFVAKQYLTIFKLSNAQNNKP
ILVQFDKVKQIRTKNGQNMAFVTLNDGIETLDGVIFPNQFKKYEELLSHNDLFIVSGKFDLRKQQRQLII
NEIQTLATFEEQKLAFAKQIIIRNKSHIDAFEEAIKATKENANDVVLSFYDETIKQMTTLGYINQKDSMF
NNFIQSFNPSDIRLI

>SAEMRSA116260 putative septation ring formation regulator
MVLYIILAIIVIILIAVGVLFYLRSNKRQIIEKAIERKNEIETLPFDQNLAQLSKLNLKGETKTKYDAMK
KDNVESTNKYLAPVEEKIHNAEALLDKFSFNAAQSEIDDANELMDSYEQSYQQQLEDVNEIIALYKDNDE
LYDKCKVDYREMKRDVLANRHQFGEAASLLETEIEKFEPRLEQYEVLKADGNYVQAHNHIAALNEQMKQL
RSYMEEIPELIRETQKELPGQFQDLKYGCRDLKVEGYDLDHVKVDSTLQSLKTELSFVEPLISRLELEEA
NDKLANINDKLDDMYDLIEHEVKAKNDVEETKDIITDNLFKAKDMNYTLQTEIEYVRENYYINESDAQSV
RQFENEIQSLISVYDDILKEMSKSAVRYSEVQDNLQYLEDHVTVINDKQEKLQNHLIQLREDEAEAEDNL
LRVQSKKEEVYRRLLASNLTSVPERFIIMKNEIDHEVRDVNEQFSERPIHVKQLKDKVSKIVIQMNTFED
EANDVLVNAVYAEKLIQYGNRYRKDYSNVDKSLNEAERLFKNNRYKRAIEIAEQALESVEPGVTKHIEEE
VIKQ

>SAI7S6 1012790 UDP-N-acetylmuramate--L-alanine ligase
MSKEFYIMTHYHFVGIKGSGMSSLAQIMHDLGHEVQGSDIENYVFTEVALRNKGIKILPFDANNIKEDMV
VIQGNAFASSHEEIVRAHQLKLDVVSYNDFLGQIIDQYTSVAVTGAHGKTSTTGLLSHVMNGDKKTSFLI
GDGTGMGLPESDYFAFEACEYRRHFLSYKPDYAIMTNIDFDHPDYFKDINDVFDAFQEMAHNVKKGIIAW
GDDEHLRKIEADVPIYYYGFKDSDDIYAQNIQITDKGTAFDVYVDGEFYDHFLSPQYGDHTVLNALAVIA
ISYLEKLDVTNIKEALETFGGVKRRFNETTIANQVIVDDYAHHPREISATIETARKKYPHKEVVAVFQPH
TFSRTQAFLNEFAESLSKADRVFLCEIFGSIRENTGALTIQDLIDKIEGASLINEDSINVLEQFDNAVVL
FMGAGDIQKLQNAYLDKLGMKNAF

>SAEMRSA116620 putative polysaccharide biosynthesis protein
MSESKEMVRGTFLITISILITKVLGVLFIIPFNYLIGGQENMAPFTYAYAPYNIAIAVATAGVPLAASKY
VAKYNAIGAYKVSQKFYKSSFIVMSITGVLGFLVLYFLAPYISELTLARNVHDKNGWSVDDITWIIRIIS
MVVIFIPVLATWRGIFQGYKSMGPTAVSEVTEQIARVIFILIGSYLVLNVFDGSILLANGIATFAAAVGA
IIGIFTLWYYWRKRKHNIDRMVESDYTDIDVSYGKMYKEIIAYSIPFVIVSLNYPLFNLVDQFTHNGALS
LVGIPSQLQDIFFNMLNMSTNKIVMIPTSLSAGFAVSLIPYITKTFAEGRLHEMHHQIRTSIGVLMFITV
PASIGIMALAQPLFTVFYGYDPIVLGHDPNHDGSRLLFYYAPVAILISLLSVTASMLQGIDKQKLTVYVI
LASVVIKLALNYPLIMLFHTPGAILSTSIALLFAIGCNFYILKKYAKFKFSYSWIHFAKIFLYSFIMMLG
VELVFFLANLFLEPTKLGYLIIIILGVTVGILIYGTITIKTRLADEFLGEIPEKLRRRVRFLR

>SAEMRSA116750 riboflavin biosynthesis protein
MQFDNIDSALMALKNGETIIVVDDENRENEGDLVAVTEWMNDNTINFMAKEARGLICAPVSKDIAQRLDL
VQMVDDNSDIFGTQFTVSIDHVDTTTGISAYERTLTAKKLIDPSSEAKDFNRPGHLFPLVAQDKGVLARN
GHTEAAVDLAKLTGAKPAGVICEIMNDDGTMAKGQDLQKFKEKHQLKMITIDDLIEYRKKLEPEIEFKAK
VKMPTDFGTFDMYGFKATYTDEEIVVLTKGAIRQHENVRLHSACLTGDIFHSQRCDCGAQLESSMKYINE
HGGMIIYLPQEGRGIGLLNKLRAYELIEQGYDTVTANLALGFDEDLRDYHIAAQILKYFNIEHINLLSNN
PSKFEGLKQYGIDIAERIEVIVPETVHNHDYMETKKIKMGHLI

>SAEMRSA116760 riboflavin synthase alpha chain
MFTGIVEEIGVVKSVQIRQSVRTIEIEAHKITADMHIGDSISVNGACLTVIDFNQTSFTVQVIKGTENKT
YLADVKRQSEVNLERAMSGNGRFGGHFVLGHVDELGTVSKINETANAKIITIQCSQHINNQLVKQGSITV
DGVSLTVFDKHDNSFDIHLIPETRRSTILSSKKLGDKVHLETDVLFKYVENILNKDKDQLSVDKLRAFGF

>SAEMRSA117370 ferrochelatase
MTKKMGLLVMAYGTPYKESDIEPYYTDIRHGKRPSEEELQDLKDRYEFIGGLSPLAGTTDDQADALVSAL
NKAYADVEFKLYLGLKHISPFIEDAVEQMHNDGITEAITVVLAPHYSSFSVGSYDKRADEEAAKYGIQLT
HVKHYYEQPKFIEYWTNKVNETLAQIPEEEHKDTVLVVSAHSLPKGLIEKNNDPYPQELEHTALLIKEQS
NIEHIAIGWQSEGNTGTPWLGPDVQDLTRDLYEKHQYKNFIYTPVGFVCEHLEVLYDNDYECKVVCDDIG
ANYYRPKMPNTHPLFIGAIVDEIKSIF

>SAEMRSA117980 conserved hypothetical protein
MHELTIYHFMSDKLNLYSDIGNIIALRQRAKKRNIKVNVVEINETEGITFDECDIFFIGGGSDREQALAT
KELSKIKTPLKEAIEDGMPGLTICGGYQFLGKKYITPDGTELEGLGILDFYTESKTNRLTGDIVIESDTF
GTIVGFENHGGRTYHDFGTLGHVTFGYGNNDEDKKEGIHYKNLLGTYLHGPILPKNYEITDYLLEKACER
KGIPFEPKEIDNEAEIQAKQVLIDRANRQKKSR

>SAI7S6 1014340 Putative PF08353 domain protein
LRNFFEVALSCYNDASGNLRRKHMRQWTAIHLAKLARKASRAVGKRGTDLPGQIARKVDTDVLRKLAEQV
DDIVFISGTNGKTTTSNLIGHTLKANNIQIIHNNEGANMAAGITSAFIMQSTPKTKIAVIEIDEGSIPRV
LKEVTPSMMVFTNFFRDQMDRFGEIDIMVNNIAETISNKGIKLLLNADDPFVSRLKIASDTIVYYGMKAH
AHEFEQSTMNESRYCPNCGRLLQYDYIHYNQIGHYHCQCGFKREQAKYEISSFDVAPFLYLNINDEKYDM
KIAGDFNAYNALAAYTVLRELGLNEQTIKNGFETYTSDNGRMQYFKKERKEAMINLAKNPAGMNASLSVG
EQLEGEKVYVISLNDNAADGRDTSWIYDADFEKLSKQQIEAIIVTGTRAEELQLRLKLAEVEVPIIVERD
IYKATAKTMDYKGFTVAIPNYTSLAPMLEQLNRSFEGGQS

>SAEMRSA118290 manganese-dependent inorganic pyrophosphatase
LTMAKTYIFGHKNPDTDAISSAIIMAEFEQLRGNSGAKAYRLGDVSAETQFALDTFNVPAPELLTDDLDG
QDVILVDHNEFQQSSDTIASATIKHVIDHHRIANFETAGPLCYRAEPVGCTATILYKMFRERGFEIKPEI
AGLMLSAIISDSLLFKSPTCTQQDVKAAEELKDIAKVDIQKYGLDMLKAGASTTDKSVEFLLNMDAKSFT
MGDYVTRIAQVNAVDLDEVLNRKEDLEKEMLAVSAQEKYDLFVLVVTDIINSDSKILVVGAEKDKVGEAF
NVQLEDDMAFLSGVVSRKKQIVPQITEALTK

>SAV2051 similar to glycoprotein endopeptidase
MNKLRRQLSMNSLLIDTSNQPLSVALMQNDEVLAEITTDSKQNHSVQLMPAISQLFEQSKIAKQQLDAII
VAEGPGSYTGLRIGVTVAKTLAYALDVKLYGVSSLKALAATIDHTDKLLVPVFDARRQAVYTGIFQWQNG
QLVTILEDQYMSIEALRAFLEDLNQPFIYIGKDTIKLQDELQGEVIAQLPNASVMYQLIDKPTDVHTFTP
KYHKLAEAERNWINSQKNN

>SAEMRSA119600 conserved hypothetical protein
MNEKHNIGESTLIKINNLDEMNQFAMFLVEQLKSGDLILLNGDLGAGKTTLTQFIGKALGVRRTINSPTF
NIIKSYRGKNLKLHHMDCYRLEDSDEDLGFDEFFEDQAITVIEWSQFIKDLLPSTHLSINISTISENTRQ
IELFAQGEHYEQIKEAIIHEFAAH

>SAI7S6 1015240 Alanine racemase
VGITGGSNMSDKYYRSAYMNVDLNAVASNFKVFSTLHPNKTVMAVVKANAYGLGSVKVARHLMENGATFF
AVATLDEAIELRMHGITAKILVLGVLPAKDIDKAIQHRVALTVPSKQWLKEAIKNISGEQEKKLWLHIKL
DTGMGRLGIKDTNTYQEVIEIIQQYEQLVFEGVFTHFACADEPGDMTTEQYQRFKDMVNEAIKPEYIHCQ
NSAGSLLMDCQFCNAIRPGISLYGYYPSEYVQQKVKVHLKPSVQLIANVVQTKTLQAGESVSYGATYTAT
DPTTIALLPIGYADGYLRIMQGSFVNVNGHQCEVIGRVCMDQTIVKVPDQVKAGDSVILIDNHRESPQSV
EVVAEKQHTINYEVLCNLSRRLPRIYHDGDQRFVTNELLK

>SAEMRSA119910 putative UDP-N-acetylmuramoylalanyl-D-glutamyl-2, 6-diaminopimelate--D-alanyl-D-alanyl ligase
MINVTLKQIQSWIPCEIEDQFLNQEINGVTIDSRAISKNMLFIPFKGENVDGHRFVSKALQDGAGAAFYQ
RGTPIDENVSGPIIWVEDTLTALQQLAQAYLRHVNPKVIAVTGSNGKTTTKDMIESVLHTEFKVKKTQGN
YNNEIGLPLTILELDNDTEISILEMGMSGFHEIEFLSNLAQPDIAVITNIGESHMQDLGSREGIAKAKSE
ITIGLKDNGTFIYDGDEPLLKPHVKEVENVKCISIGVAADNALVCSVDDRDTTGISFTINNKEHYDLPIL
GKHNMKNATIAIAVGHELGLTYNTIYQNLKNVSLTGMRMEQHTLENDITVINDAYNASPTSMRAAIDTLS
TLTGRRILILGDVLELGENSKEMHISVGNYLEEKHIDVLYTFGNEAKYIYDSGQQHVEKAQHFNSKDDMI
EVLTSDLKAHDRVLVKGSRGMKLEEVVNALIS

>SAI7S6 1015360 D-alanine--D-alanine ligase
VILLRFKDANKSINNRTKSILIYIKVANPDISLEENEMTKENICIVFGGKSAEHEVSILTAQNVLNAIDK
DKYHVDIIYITNDGDWRKQNNITAEIKSTDELHLENGEALEISQLLKESSSGQPYDAVFPLLHGPNGEDG
TIQGLFEVLDVPYVGNGVLSAASSMDKLVMKQLFEHRGLPQLPYISFLRSEYEKYEHNILKLVNDKLNYP
VFVKPANLGSSVGISKCNNEAELKEGIKEAFQFDRKLVIEQGVNAREIEVAVLGNDYPEATWPGEVVKDV
AFYDYKSKYKDGKVQLQIPADLDEDVQLTLRNMALEAFKATDCSGLVRADFFVTEDNQIYINETNAMPGF
TAFSMYPKLWENMGLSYPELITKLIELAKERHQDKQKNKYKID

>SAEMRSA119990 putative membrane protein
MKKKALLPLFLGIMVFLAGCDYSKPEKRSGFFYNTFVDPMKNVLDWLGNNLLNDNYGLAIIILVLVIRII
LLPFMLSNYKNSHMMRQKMKVAKPEVEKIQEKVKRARTQEEKMAANQELMQVYKKYDMNPIKSMLGCLPM
LIQLPIIMGLYFVLKDQLVDGLFKYPHFLWFDLGRPDIWITIIAGVLYFIQAYVSSKTMPDEQRQMGYMM
MVISPIMIIWISLSSASALGLYWSVSAAFLVVQTHFANIYYEKVAKKEVQPFIEAYEREHNGGSNKKGKN
TQVVSKKKKK

>SAEMRSA120080 putative UDP-N-acetylglucosamine 1-carboxyvinyltransferase
MDKIVIKGGNKLTGEVKVEGAKNAVLPILTASLLASDKPSKLVNVPALSDVETINNVLTTLNADVTYKKD
ENAVVVDATKTLNEEAPYEYVSKMRASILVMGPLLARLGHAIVALPGGCAIGSRPIEQHIKGFEALGAEI

HLENGNIYANAKDGLKGTSIHLDFPSVGATQNIIMAASLAKGKTLIENAAKEPEIVDLANYINEMGGRIT
GAGTDTITINGVESLHGVEHAIIPDRIEAGTLLIAGAITRGDIFVRGAIKEHMASLVYKLEEMGVELDYQ
EDGIRVRAEGELQPVDIKTLPHPGFPTDMQSQMMALLLTANGHKVVTETVFENRFMHVAEFKRMNANINV
EGRSAKLEGKSQLQGAQVKATDLRAAAALILAGLVADGKTSVTELTHLDRGYVDLHGKLKQLGADIERIN
D

>SAEMRSA120140 putative ATP synthase delta chain
MVKVANKYAKALFDVSLDTNNLETINQELTVINEAVKDKIEQLKMVDSNPTQTAEQRRELINGVFTDINP
YIKNMMYVLADNRHISLIADVFKAFQSLYNGHYNQDFATIESTYELSQEELDKIVKLVTQQTKLSKVIVD
TKINPDLIGGFRVKVGTTVLDGSVRNDLVQLQRKFRRVN

>SAEMRSA120150 putative ATP synthase subunit b
VTETANLFVLGAAGGVEWGTVIVQVLTFIVLLALLKKFAWGPLKDVMDKRERDINRDIDDAEQAKLNAQK
LEEENKQKLKETQEEVQKILEDAKVQARQQQEQIIHEANVRANGMIETAQSEINSQKERAIADINNQVSE
LSVLIASKVLRKEISEQDQKALVDKYLKEAGDK

>SAEMRSA120170 ATP synthase subunit a
MDHKSPLVSWNLFGFDIVFNLSSILMILVTAFLVFLLAIICTRNLKKRPTGKQNFVEWIFDFVRGIIEGN
MAWKKGGQFHFLAVTLILYIFIANMLGLPFSIVTKDHTLWWKSPTADATVTLTLSTTIILLTHFYGIKMR
GTKQYLKGYVQPFWPLAIINVFEEFTSTLTLGLRLYGNIFAGEILLTLLAGLFFNEPAWGWIISIPGLIV
WQAFSIFVGTIQAYIFIMLSMVYMSHKVADEH

>SAEMRSA120330 putative tagatose-bisphosphate aldolase
MPLVSMKEMLIDAKENGYAVGQYNINNLEFTQAILEASQEENAPVILGVSEGAARYMSGFYTIVKMVEGL
MHDLNITIPVAIHLDHGSSFEKCKEAIDAGFTSVMIDASHSPFEENVATTKKVVEYAHEKGVSVEAELGT
VGGQEDDVVADGIIYADPKECQELVEKTGIDALAPALGSVHGPYKGEPKLGFKEMEEIGLSTGLPLVLHG
GTGIPTKDIQKAIPFGTAKINVNTENQIASAKAVRDVLNNDKEVYDPRKYLGPAREAIKETVKGKIKEFG
TSNRAK

>SAEMRSA120690 putative membrane protein
MDFSNFFQNLSTLKIVTSILDLLIVWYVLYLLITVFKGTKAIQLLKGILVIVIGQQISMILNLTATSKLF
DIVIQWGVLALIVIFQPEIRRALEQLGRGSFLKRYTSNTYSKDEEKLIQSVSKAVQYMAKRRIGALIVFE
KETGLQDYIETGIAMDSNISQELLINVFIPNTPLHDGAMIIQGTKIAAAASYLPLSDSPKISKSLGTRHR
AAVGISEVSDAFTVIVSEETGDISVTFDGKLRRDISNEIFEELLAEHWFGTRFQKKGVK

>SAEMRSA121190 putative cobalt transport protein
MKNKLIIGRYLPINSFVHHLDPRAKLMFVFLFIILIFFCHSPLTYLWVFALILFFMRLAKIQLWFLIKGL
TPIFFFLIFTLMMHIFLTKGGYVLVEWHGITIETNGILEGLYISLRLIGIVMIATIMTLSTSPIDLTDAF
ERLLAPLKMFKLPVHQLSMIMSIALRFIPTLMDELDKIILAQKSRGSEISSGNIATRIKSFIPLLVPLFI
SAFQRAEELAVAMEVRGYDANVQRTSYRQLKWQLRDTLSLIMIIPFAIILFVLKYSGV

>SAEMRSA121290 preprotein translocase SecY subunit
MIQTLVNFFRTKEVRNKIFFTLAMLVIFKIGTYIPAPGVNPAAFDNPQGSQGATELLNTFGGGALKRFSI
FAMGIVPYITASIVMQLLQMDIVPKFSEWAKQGEVGRRKLNNVTRYLAISLAFIQSIGMAFQFNNYLKGA
LIINQSIMSYLLIALVLTAGTAFLIWLGDQITQFGVGNGISIIIFAGILSTLPASLIQFGQTAFVGQEDT
SLAWLKVLGLLVSLILLTVGAIYVLEAVRKIPIQYAKKQTAQRLGSQATYLPLKVNSAGVIPVIFAMAFF
LLPRTLTLFYPDKEWAQNIANAANPSSNVGMVVYIVLIILFTYFYAFVQVNPEKMADNLKKQGSYVPGIR
PGEQTKKYITKVLYRLTFVGSIFLAVISILPILATKFMGLPQSIQIGGTSLLIVIGVAIETMKSLEAQVS
QKEYKGFGGR

>SAEMRSA121370 50S ribosomal protein L5
LNRLKEKFNTEVTENLMKKFNYSSVMEVPKIDKIVVNMGVGDAVQNSKVLDNAVEELELITGQKPLVTKA
KKSIATFRLREGMPIGAKVTLRGERMYEFLDKLISVSLPRVRDFQGVSKKAFDGRGNYTLGVKEQLIFPE
IDYDKVSKVRGMDIVIVTTANTDEEARELLANFGMPFRK

>SAEMRSA121430 30S ribosomal protein S3
VGQKINPIGLRVGIIRDWEAKWYAEKDFASLLHEDLKIRKFIDNELKEASVSHVEIERAANRINIAIHTG
KPGMVIGKGGSEIEKLRNKLNALTDKKVHINVIEIKKVDLDARLVAENIARQLENRASFRRVQKQAITRA
MKLGAKGIKTQVSGRLGGADIARAEQYSEGTVPLHTLRADIDYAHAEADTTYGKLGVKVWIYRGEVLPTK
NTSGGGK

>SAEMRSA125010 putative pantoate--beta-alanine ligase
MTKLITTVKEMQHIVKAAKRSGTTIGFIPTMGALHDGHLTMVRESVSTNDITVVSVFVNPLQFGPNEDFD
AYPRQIDKDLELVSEVGADIVFHPAVEDMYPGELGIDVKVGPLADVLEGAKRPGHFDGVVTVVNKLFNIV
MPDYAYFGKKDAQQLAIVEQMVKDFNHAVEIIGIDIVREADGLAKSSRNVYLTEQERQEAVHLSKSLLLA
QALYQDGERQSKVIIDRVTEYLESHISGRIEEVAVYSYPQLVEQHEITGRIFISLAVKFSKARLIDNIII
GAE

>SAEMRSA125020 putative 3-methyl-2-oxobutanoatehydroxymethyltransferase
LKTVSQLIDMKQKQTKISMVTAYDFPSAKQVEAAGIDMILVGDSLGMTVLGYESTVQVTLADMIHHGRAV
RRGAPNTFVVVDMPIGAVGISMTQDLNHALKLYQETNANAIKAEGAHITPFIEKATAIGIPVVAHLGLTP
QSVGVMGYKLQGATKEAAEQLILDAKNVEQAGAVALVLEAIPNDLAEEISKHLTIPVIGIGAGKGTDGQV
LVYHDMLNYGVEHKAKFVKQFADFSVGVDGLKQYDQEVKSGAFPSEEYTYKKKIMNEVNNND

>SAI7S6 1018940 Hydroxymethylglutaryl-CoA reductase
MSMQSLDKNFRHLSRQQKLQQLVDKQWLSEDQFDILLNHPLIDEEVANSLIENVIAQGALPVGLLPNIIV
DDKAYVVPMMVEEPSVVAAASYGAKLVNQTGGFKTVSSERIMIGQIVFDGVDDTEKLSADIKALEKQIHK
IADEAYPSIKARGGGYQRIAIDTFPEQQLLSLKVFVDTKDAMGANMLNTILEAITAFLKNESPQSDILMS
ILSNHATASVVKVQGEIDVKDLARGERTGEEVAKRMERASVLAQVDIHRAATHNKGVMNGIHAVVLATGN
DTRGAEASAHAYASRDGQYRGIATWRYDQKRQRLIGTIEVPMTLAIVGGGTKVLPIAKASLELLNVDSAQ
ELGHVVAAVGLAQNFAACRALVSEGIQQGHMSLQYKSLAIVVGAKGDEIAQVAEALKQEPRANTQVAERI
LQEIRQQ

>SAEMRSA121230 DNA-directed RNA polymerase alpha chain
MIEIEKPRIETIEISEDAKFGKFVVEPLERGYGTTLGNSLRRILLSSLPGAAVKYIEIEGVLHEFSAVDN
VVEDVSTIIMNIKQLALKIYSEEDKTLEIDVRDEGEVTASDITHDSDVEILNPELKIATVSKGGHLKIRL
VANKGRGYALAEQNNTSDLPIGVIPVDSLYSPVERVNYTVENTRVGQSSDFDKLTLDVWTNGSITPQESV
SLAAKIMTEHLNIFVGLTDEAQNAEIMIEKEEDQKEKVLEMSIEELDLSVRSYNCLKRAGINSVQELADK
SEADMMKVRNLGRKSLEEVKYKLEDLGLGLRKED

>RK77 010475threonylcarbamoyl-AMP synthase
VHSFLLYIMINKKGVIKLDTKIWDVREYNEDLQQYPKINEIKDIVLNGGLIGLPTETVYGLAANATDEEA
VAKIYEAKGRPSDNPLIVHIHSKDQLKDFTYILDAPVEKLMQAFWPGPISFILPLKPGYLCRKVSGGLSS
VAVRMPSHSVGRQLLQIINEPLAAPSANLSGRPSPTTFNHVYQDLNGRIDGIVQAEQSEEGLESTVLDCT
SFPYKIARPGSITAAMITDILPNSITHADYNDTEQPIAPGMKYKHYSPNTPLTIITDIESKIGNDGKDWS
SIAFIVPSNKVAFIPSEAQFIQLCQDDNDVKQASHNLYDVLHSLDENENISAAYIYGFELNDNTEAIMNR
MLKAAGNHIVKGCEL

>SAEMRSA115480 histidyl-tRNA synthetase
MIKIPRGTQDILPEDSKKWRYIENQLDELMTFYNYKEIRTPIFESTDLFARGVGDSTDVVQKEMYTFKDK
GDRSITLRPEGTAAVVRSYIEHKMQGNPNQPIKLYYNGPMFRYERKQKGRYRQFNQFGVEAIGAENPSVD
AEVLAMVMHIYQSFGLKHLKLVINSVGDMASRKEYNEALVKHFEPVIHEFCSDCQSRLHTNPMRILDCKV
DRDKEAIKTAPRITDFLNEESKAYYEQVKAYLDDLGIPYIEDPNLVRGLDYYTHTAFELMMDNPNYDGAI
TTLCGGGRYNGLLELLDGPSETGIGFALSIERLLLALEEEGIELDIEENLDLFIVTMGDQADRYAVKLLN
HLRHNGIKADKDYLQRKIKGQMKQADRLGAKFTIVIGDQELENNKIDVKNMTTGESETIELDALVEYFKK

>SAEMRSA112890 dihydrofolate reductase type I
MTLSILVAHDLQRVIGFENQLPWHLPNDLKHVKKLSTGHTLVMGRKTFESIGKPLPNRRNVVLTSDTSFN

VEGVDVIHSIEDIYQLPGHVFIFGGQTLFEEMIDKVDDMYITAIEGKFRGDTFFPPYTFEDWEVTSSVEG
KLDEKNTIPHTFLHLIRKK

>SAEMRSA110440 putative flavoprotein
MKKILLAVTGGIAAYKAIDLTSKLTQSGYEVRVMLTNHAQKFVTPLAFQAISRNAVYTDTFIEENPSEIQ
HIALGDWADAIIVAPATANTIAKLSVGIADDLVTSTLLATETPKFIAPAMNVHMYENKRTQQNINILKED
GYHFIEPGSGFLACGYVAKGRMEEPLQIVSVIDAHFQNSNRLANSSFQDKRALVTAGPTIEVIDPVRFVS
NRSSGKMGYAIAEALRNRGAIVTLVAGPTTLEDPKDIEVIHVQSAEEMFEQVTSRFDEQDIVVKAAAVSD
YTPVDVLEHKMKKQDGDLSVSFKRTKDILKYLGEHKTSQYLIGFAAETEDIENYAQQKLRKKNADVIISN
NVGDMSIGFSSDDNELTMHFKNNEKVNIKKGKKVVLAAQILDELETRWQ

Sequence 2: Non_cytoplasmic_localized_proteins_(PSE_MEM_proteins)

>SAEMRSA106090_putative_undecaprenol_kinase
MFIIELIKGIILGVVEGLTEFAPVSSTGHMILVDDMWLKSSEFLGSQSAFTFKIVIQLGSVFAAAWVFRE
RFLEILHIGKHKHVEGENDQQRRSKPRRLNLLHVLVGMVPAGILGLLFDDFIEEHLFSVPTVMIGLFVGA
IYMIIADKYSAKVKNPQTVDQINYFQAFVIGISQAVAMWPGFSRSGSTISTGVLMKLNHKAASDFTFIMA
VPIMLAASGLSLLKHYQDIQIADIPFYILGFLAAFTVGLIAIKTFLHLINKIKLIPFAIYRIVLVIFIAI
LYFGFGIGKGI

>SAEMRSA106730_putative_glycosyltransferase
MVTLLLVAVTMIVSLTITPIVIAISKRLNLVDKPNFRKVHTKPISVMGGTVILFSFLIGIWIGHPIETEI
KPLIIGAIIMYVLGLVDDIYDLKPYIKLAGQIAAALVVAFYGVTIDFISLPMGTTIHFGFLSIPITVIWI
VAITNAINLIDGLDGLASGVSAIGLITIGFIAILQANIFITMICCVLLGSLIGFLFYNFHPAKIFLGDSG
ALMIGFIIGFLSLLGFKNITIIALFFPIVILAVPFIDTLFAMIRRVKKGQHIMQADKSHLHHKLLALGYT
HRQTVLLIYSISILFSLSSIILYVSPPLGVVLMFILIIFSIELIVEFTGLIDNNYRPILNLISRKSSHKE
D

>SAEMRSA110140_penicillin-binding_protein_1
MAKQKIKIKKNKIGAVLLVGLFGLLFFILVLRISYIMITGHSNGQDLVMKANEKYLVKNAQQPERGKIYD
RNGKVLAEDVERYKLVAVIDKKASANSKKPRHVVDKKETAKKLSTVIDMKPEEIEKRLSQKKAFQIEFGR
KGTNLTYQDKLKIEKMNLPGISLLPETERFYPNGNFASHLIGRAQKNPDTGELKGALGVEKIFDSYLSGS
KGSLRYIHDIWGYIAPNTKKEKQPKRGDDVHLTIDSNIQVFVEEALDGMVERYQPKDLFAVVMDAKTGEI
LAYSQRPTFNPETGKDFGKKWANDLYQNTYEPGSTFKSYGLAAAIQEGAFDPDKKYKSGHRDIMGSRISD
WNRVGWGEIPMSLGFTYSSNTLMMHLQDLVGADKMKSWYERFGFGKSTKGMFDGEAPGQIGWSNELQQKT
SSFGQSTTVTPVQMLQAQSAFFNDGNMLKPWFVNSVENPVSKRQFYKGQKQIAGKPITKDTAEKVEKQLD
LVVNSKKSHAANYRIDGYEVEGKTGTAQVAAPNGGGYVKGPNPYFVSFMGDAPKKNPKVIVYAGMSLAQK
NDQEAYELGVSKAFKPIMENTLKYLNVGKSKDDTSNAEYSKVPDVEGQDKQKAIDNVSAKSLEPVTIGTG
TQIKAQSIKAGNKVLPHSKVLLLTDGDLTMPDMTGWTKEDVIAFENLTNIKVNLKGSGFVSHQSISKGQK
LTEKDKIDVEFSSENVDSNSTNNSDSNSDDKKKSDSKTDKDKSD

>SAEMRSA110150_phospho-N-acetylmuramoyl-pentapeptide-transfera_se
MIFVYALLALVITFVLVPVLIPTLKRMKFGQSIREEGPQSHMKKTGTPTMGGLTFLLSIVITSLVAIIFV
DQANPIILLLFVTIGFGLIGFIDDYIIVVKKNNQGLTSKQKFLAQIGIAIIFFVLSNVFHLVNFSTSIHI
PFTNVAIPLSFAYVIFIVFWQVGFSNAVNLTDGLDGLATGLSIIGFTMYAIMSFVLGETAIGIFCIIMLF
ALLGFLPYNINPAKVFMGDTGSLALGGIFATISIMLNQELSLIFIGLVFVIETLSVMLQVASFKLTGKRI
FKMSPIHHHFELIGWSEWKVVTVFWAVGLISGLIGLWIGVH

>SAEMRSA110290_lipoprotein_signal_peptidase
MHKKYFIGTSILIAVFVVIFDQVTKYIIATTMKIGDSFEVIPHFLNITSHRNNGAAWGILSGKMTFFFII
TIIILIALVYFFIKDAQYNLFMQVAISLLFAGALGNFIDRILTGEVVDFIDTNIFGYDFPIFNIADSSLT

IGVILIIIALLKDTSNKKEKEVK

>SAEMRSA112010_putative_membrane_protein
MMIIVMLLLSYLIGAFPSGFVIGKLFFKKDIRQFGSGNTGATNSFRVLGRPAGFLVTFLDIFKGFITVFF
PLWLPVHADGPISTFFTNGLIVGLCAILGHVYPVYLKFQGGKAVATSAGVVLGVNPILLLILAIIFFVVL
KIFKYVSLASIVAAICCVIGSLIIQDYILLVVSFLVSIILIIRHRSNIARIFRGEEPKIKWM

>SAEMRSA112810_putative_UDP-N-acetylglucosamine-N-acetylmuramyl-(pentapeptide)pyr_ophosphoryl-undecaprenol_N-acetylglucosamine_transferase
MKKIAFTGGGTVGHVSVNLSLIPTALSQGYEALYIGSKNGIEREMIESQLPEIKYYPISSGKLRRYISLE
NAKDVFKVLKGILDARKVLKKEKPDLLFSKGGFVSVPVVIAAKSLNIPTIIHESDLTPGLANKIALKFAK
KIYTTFEETLNYLPKEKADFIGATIREDLKNGNAHNGYQLTGFNENKKVLLVMGGSLGSKKLNSIIRENL
DALLQQYQVIHLTGKGLKDAQVKKSGYIQYEFVKEELTDLLAITDTVISRAGSNAIYEFLTLRIPMLLVP
LGLDQSRGDQIDNANHFADKGYAKAIDEEQLTAQILLQELNEMEQERTRIINNMKSYEQSYTKEALFDKM
IKDALN

>SAEMRSA113110_penicillin-binding_protein_2
MTENKGSSQPKKNGNNGGKSNSKKNRNVKRTIIKIIGFMIIAFFVVLLLGILLFAYYAWKAPAFTEAKLQ
DPIPAKIYDKNGELVKTLDNGQRHEHVNLKDVPKSMKDAVLATEDNRFYEHGALDYKRLFGAIGKNLTGG
FGSEGASTLTQQVVKDAFLSQHKSIGRKAQEAYLSYRLEQEYSKDDIFQVYLNKIYYSDGVTGIKAAAKY
YFNKDLKDLNLAEEAYLAGLPQVPNNYNIYDHPKAAEDRKNTVLYLMHYHKRITDKQWEDAKKIDLKANL
VNRTAEERQNIDTNQDSEYNSYVNFVKSELMNNKAFKDENLGNVLQSGIKIYTNMDKDVQKTLQNDVDNG
SFYKNKDQQVGATILDSKTGGLVAISGGRDFKDVVNRNQATDPHPTGSSLKPFLAYGPAIENMKWATNHA
IQDESSYQVDGSTFRNYDVKSHGTVSIYDALRQSFNIPALKAWQSVKQNAGNDAPKKFAAKLGLNYEGDI
GPSEVLGGSASEFSPTQLASAFAAIANGGTYNNAHSIQKVVTRDGETIEYDHTSHKAMSDYTAYMLAEML
KGTFKPYGSAYGHGVSGVNMGAKTGTGTYGAETYSQYNLPDNAAKDVWINGFTPQYTMSVWMGFSKVKQY
GENSFVGHSQQEYPQFLYENVMSKISSRDGEDFKRPSSVSGSIPSINVSGSQDNNTTNRSAHGGSDTSAN
SSGTAQSNNNTRSQQSRNSGGLTGIFN

>SAEMRSA115170_conserved_hypothetical_protein
MGLVRKFFMPNSYVQSIFQIDLDKLVDKGVKGIITDLDNTLVGWDVKEPTERVKAWFKEANEKGITITIV
SNNNESRVASFSQHLDIDFIFKARKPMGKAFDKAITKMNIRPDQTVVIGDQMLTDVFGGNRRGLYTIMVV
PVKRTDGFITKFNRLIERRLLRHFSKKGYITWEEN

>SAEMRSA115540_putative_protein-export_membrane_protein
VKKSSRIIAFLLLVVLLFAGMAATYKSVIKNVNLGLDLQGGFEVLYQVDPLNKGDKIDKKALQSTAQTLE
NRVNVLGVSEPKIQVEEPNRIRVQLAGVTDQNEARKILSSQANLTIRDAEDKVKLSGSDIKQGSAKQEFK
QETNQPTVTFKVKDKNKFKKVTEEISKKRDNVMVVWLDFKKGDSYKKEAQKKNPKFISAASVDQPINSDS
VEISGGFKGQEGVKKAKQIAELLNAGSLPVDLKEIYSNSVGAQFGQDALDKTVFASFIGVALIYLFMLGF
YRLPGLVAIIALTTYIYLTLVAFNFISGVLTLPGLAALVLGVGMAVDANIIMYERIKDELRIGRTIKQAF
SKANKSSFLTIFDSNLTTVIAAAVLFFFGESSVKGFATMLLLGILMIFVTAVFLSRFLLSLLVSSNIFKN
QFWLFGVKKNKRHDINEGVDVHDLKTSFEKWNFVKLAKPLIGVSILIVVVGLVILYIFKLNLGIDFSSGT
RVDFQSKQAITQQKVEQVVKDSGLKADQIQINGKDNKVATVQFKDDLTRAQDNKLSDNIKSKFGDTPQIN
TVSPIIGQELAKNAMLALIYASIGIIIYVSLRFEWRMGLSSVLALLHDVFIIVAIFSLFRIEVDLTFIAA
VLTIVGYSINDTIVTFDRVRENLQKVKVITTTEQIDDIVNRSIRQTMTRSINTVLTVIVVVVAILFFGAP
TIFNFTLALFIGLISGVFSSIFIAVPLWGIMKKRQLKKSPKHKLVVYKEKKSNDEKILV

>SAEMRSA116620_putative_polysaccharide_biosynthesis_protein
MSESKEMVRGTFLITISILITKVLGVLFIIPFNYLIGGQENMAPFTYAYAPYNIAIAVATAGVPLAASKY
VAKYNAIGAYKVSQKFYKSSFIVMSITGVLGFLVLYFLAPYISELTLARNVHDKNGWSVDDITWIIRIIS
MVVIFIPVLATWRGIFQGYKSMGPTAVSEVTEQIARVIFILIGSYLVLNVFDGSILLANGIATFAAAVGA
IIGIFTLWYYWRKRKHNIDRMVESDYTDIDVSYGKMYKEIIAYSIPFVIVSLNYPLFNLVDQFTHNGALS
LVGIPSQLQDIFFNMLNMSTNKIVMIPTSLSAGFAVSLIPYITKTFAEGRLHEMHHQIRTSIGVLMFITV
PASIGIMALAQPLFTVFYGYDPIVLGHDPNHDGSRLLFYYAPVAILISLLSVTASMLQGIDKQKLTVYVI

LASVVIKLALNYPLIMLFHTPGAILSTSIALLFAIGCNFYILKKYAKFKFSYSWIHFAKIFLYSFIMMLG
VELVFFLANLFLEPTKLGYLIIIILGVTVGILIYGTITIKTRLADEFLGEIPEKLRRRVRFLR

>SAEMRSA117460_putative_peptidyl-prolyl_cis-isomerase
MKMINKLIVPVTASALLLGACGASATDSKENTLISSKAGDVTVADTMKKIGKDQIANASFTEMLNKILAD
KYKNKVNDKKIDEQIEKMQKQYGGKDKFEKALQQQGLTADKYKENLRTAAYHKELLSDKIKISDSEIKED
SKKASHILIKVKSKKSDKEGLDDKEAKQKAEEIQKEVSKDPSKFGEIAKKESMDTGSAKKDGELGYVLKG
QTDKDFEKALFKLKDGEVSDVVKSSFGYHIIKADKPTDFNSEKQSLKEKLVDQKVQKNPKLLTDAYKDLL
KEYDVDFKDRDIKSVVEDKILNPEKLKQGGAQGGQSGMSQ

>SAEMRSA119930_putative_membrane_protein
MNYSSRQQPDKHWLRKVDWVLVATIAVLAIFSVLLINSAMGGGQYSANFGIRQIFYYILGAIFAGIIMFI
SPKKIKHYTYLLYFLICLLLIGLLVIPESPITPIINGAKSWYTFGPISIQPSEFMKIILILALARVVSRH
NQFTFNKSFQSDLLLFFKIIGVSLVPSILILLQNDLGTTLVLAAIIAGVMLVSGITWRILAPIFITGIVG
AMTVILGILYAPSLIENLLGVQLYQMGRINSWLDPYTYSSGDGYHLTESLKAIGSGQLLGKGYNHGEVYI
PENHTDFIFSVIGEELGFIGSVILILIFLFLIFHLIRLAAKIEDQFNKIFIVGFVTLLVFHILQNIGMTI
QLLPITGIPLPFISYGGSALWSMMTGIGIVLSIYYHEPKRYVDLYHPKSN

>SAEMRSA119990_putative_membrane_protein
MKKKALLPLFLGIMVFLAGCDYSKPEKRSGFFYNTFVDPMKNVLDWLGNNLLNDNYGLAIIILVLVIRII
LLPFMLSNYKNSHMMRQKMKVAKPEVEKIQEKVKRARTQEEKMAANQELMQVYKKYDMNPIKSMLGCLPM
LIQLPIIMGLYFVLKDQLVDGLFKYPHFLWFDLGRPDIWITIIAGVLYFIQAYVSSKTMPDEQRQMGYMM
MVISPIMIIWISLSSASALGLYWSVSAAFLVVQTHFANIYYEKVAKKEVQPFIEAYEREHNGGSNKKGKN
TQVVSKKKKK

>SAEMRSA120150_putative_ATP_synthase_subunit_b
VTETANLFVLGAAGGVEWGTVIVQVLTFIVLLALLKKFAWGPLKDVMDKRERDINRDIDDAEQAKLNAQK
LEEENKQKLKETQEEVQKILEDAKVQARQQQEQIIHEANVRANGMIETAQSEINSQKERAIADINNQVSE
LSVLIASKVLRKEISEQDQKALVDKYLKEAGDK

>SAEMRSA120170_ATP_synthase_subunit_a
MDHKSPLVSWNLFGFDIVFNLSSILMILVTAFLVFLLAIICTRNLKKRPTGKQNFVEWIFDFVRGIIEGN
MAWKKGGQFHFLAVTLILYIFIANMLGLPFSIVTKDHTLWWKSPTADATVTLTLSTTIILLTHFYGIKMR
GTKQYLKGYVQPFWPLAIINVFEEFTSTLTLGLRLYGNIFAGEILLTLLAGLFFNEPAWGWIISIPGLIV
WQAFSIFVGTIQAYIFIMLSMVYMSHKVADEH

>SAEMRSA120690_putative_membrane_protein
MDFSNFFQNLSTLKIVTSILDLLIVWYVLYLLITVFKGTKAIQLLKGILVIVIGQQISMILNLTATSKLF
DIVIQWGVLALIVIFQPEIRRALEQLGRGSFLKRYTSNTYSKDEEKLIQSVSKAVQYMAKRRIGALIVFE
KETGLQDYIETGIAMDSNISQELLINVFIPNTPLHDGAMIIQGTKIAAAASYLPLSDSPKISKSLGTRHR
AAVGISEVSDAFTVIVSEETGDISVTFDGKLRRDISNEIFEELLAEHWFGTRFQKKGVK

>SAEMRSA121190_putative_cobalt_transport_protein
MKNKLIIGRYLPINSFVHHLDPRAKLMFVFLFIILIFFCHSPLTYLWVFALILFFMRLAKIQLWFLIKGL
TPIFFFLIFTLMMHIFLTKGGYVLVEWHGITIETNGILEGLYISLRLIGIVMIATIMTLSTSPIDLTDAF
ERLLAPLKMFKLPVHQLSMIMSIALRFIPTLMDELDKIILAQKSRGSEISSGNIATRIKSFIPLLVPLFI
SAFQRAEELAVAMEVRGYDANVQRTSYRQLKWQLRDTLSLIMIIPFAIILFVLKYSGV

>SAEMRSA121290_preprotein_translocase_SecY_subunit
MIQTLVNFFRTKEVRNKIFFTLAMLVIFKIGTYIPAPGVNPAAFDNPQGSQGATELLNTFGGGALKRFSI
FAMGIVPYITASIVMQLLQMDIVPKFSEWAKQGEVGRRKLNNVTRYLAISLAFIQSIGMAFQFNNYLKGA
LIINQSIMSYLLIALVLTAGTAFLIWLGDQITQFGVGNGISIIIFAGILSTLPASLIQFGQTAFVGQEDT
SLAWLKVLGLLVSLILLTVGAIYVLEAVRKIPIQYAKKQTAQRLGSQATYLPLKVNSAGVIPVIFAMAFF
LLPRTLTLFYPDKEWAQNIANAANPSSNVGMVVYIVLIILFTYFYAFVQVNPEKMADNLKKQGSYVPGIR

PGEQTKKYITKVLYRLTFVGSIFLAVISILPILATKFMGLPQSIQIGGTSLLIVIGVAIETMKSLEAQVS
QKEYKGFGGR
>SAR081prolipoprotein_diacylglyceryl_transferase
MGGIVFNYIDPVAFNLGPLSVRWYGIIIAVGILLGYFVAQRALVKAGLHKDTLVDIIFYSALFGFIAARI
YFVIFQWPYYAENPGEIIKIWHGGIAIHGGLIGGFIAGVIVCKVKNLNPFQIGDIVAPSIILAQGIGRWG
NFMNHEAHGGPVSRAFLEQLHLPNFIIENMYINGQYYHPTFLYESIWDVAGFIILVNIRKHLKLGETFFL
YLTWYSIGRFFIEGLRTDSLMLTSNIRVAQLVSILLILISISLIVYRRIKYNPPLYSKVGALPWPTRKVK
>EssA
MLMNSVIALTFLTASSNNGGLNIDVQQEEEKRINNDLNQYDTTLFNKDSKAVNDAIAKQKKERQQQIKND
MFQNQASHSTRLNETKKVLFSKSNLEKTSESDKSPYIQNKQEKKIFPYILMSVGAFLTLGFVIFSIHKGR
RTKNESARKSNI
